# Genetics and Epigenetics of Gastroenteropancreatic Neuroendocrine Neoplasms

**DOI:** 10.1210/er.2018-00160

**Published:** 2019-01-17

**Authors:** Andrea Mafficini, Aldo Scarpa

**Affiliations:** 1 ARC-Net Center for Applied Research on Cancer, University and Hospital Trust of Verona, Verona, Italy; 2 Department of Diagnostics and Public Health, Section of Pathology, University and Hospital Trust of Verona, Verona, Italy

## Abstract

Gastroenteropancreatic (GEP) neuroendocrine neoplasms (NENs) are heterogeneous regarding site of origin, biological behavior, and malignant potential. There has been a rapid increase in data publication during the last 10 years, mainly driven by high-throughput studies on pancreatic and small intestinal neuroendocrine tumors (NETs). This review summarizes the present knowledge on genetic and epigenetic alterations. We integrated the available information from each compartment to give a pathway-based overview. This provided a summary of the critical alterations sustaining neoplastic cells. It also highlighted similarities and differences across anatomical locations and points that need further investigation. GEP-NENs include well-differentiated NETs and poorly differentiated neuroendocrine carcinomas (NECs). NENs are graded as G1, G2, or G3 based on mitotic count and/or Ki-67 labeling index, NECs are G3 by definition. The distinction between NETs and NECs is also linked to their genetic background, as *TP53* and *RB1* inactivation in NECs set them apart from NETs. A large number of genetic and epigenetic alterations have been reported. Recurrent changes have been traced back to a reduced number of core pathways, including DNA damage repair, cell cycle regulation, and phosphatidylinositol 3-kinase/mammalian target of rapamycin signaling. In pancreatic tumors, chromatin remodeling/histone methylation and telomere alteration are also affected. However, also owing to the paucity of disease models, further research is necessary to fully integrate and functionalize data on deregulated pathways to recapitulate the large heterogeneity of behaviors displayed by these tumors. This is expected to impact diagnostics, prognostic stratification, and planning of personalized therapy.

**Essential Points**

- Gastroenteropancreatic neuroendocrine neoplasms are rare and heterogeneous as for anatomical site, biological features, prognosis, and therapeutic options
- Gastroenteropancreatic neuroendocrine tumors are a biologically different entity from the more aggressive neuroendocrine carcinomas, as recently underlined by the 2017 World Health Organization classification
- Genetics and epigenetics information is relatively abundant for pancreatic and ileal neuroendocrine tumors, whereas it is very limited for the other anatomical sites
- Genetic syndromes gave many insights into pancreatic endocrine tumors biology, whereas their relationship with ileal neuroendocrine tumors is less defined
- Recent genomics and epigenomics studies provided a first level of integration of biological data, showing the convergence of different alterations into a limited number of pathways
- The mammalian target of rapamycin pathway and cell cycle dysregulation appear as a common feature of ileal and pancreatic neuroendocrine tumors, achieved by different mechanisms and with different modulation effects and therapeutic implications
- Further integration of high-throughput genetic and epigenetic analysis is necessary to enable informed precision therapy, although the relevance of the achieved information for the other anatomical sites should be assessed

Gastroenteropancreatic (GEP) neuroendocrine neoplasms (NENs) are relatively rare (1 and 3.5 new cases per year per 100,000 individuals in Europe and the United States, respectively), but their incidence rate has more than tripled in the last 40 years (1–4). GEP-NENs include well-differentiated neuroendocrine tumors (NETs) and poorly differentiated neuroendocrine carcinomas (NECs). NETs are graded as grade 1 (G1), grade 2 (G2), or grade 3 (G3) based on mitotic count and/or Ki-67 labeling index; NECs are G3 by definition.

GEP-NENs were discovered in 1907 by Siegfried Oberdorfer (5), who further described their malignant potential in 1929 (6). He named them “carcinoids” to distinguish them from the more aggressive carcinomas. The original concept of carcinoids as benign or indolent neoplasms progressively left a place for the idea of variable behavior (7). This culminated in the 2010 World Health Organization (WHO) classification of tumors of the digestive system: all GEP-NETs were defined as potentially malignant, albeit with varying degrees (8).

Heterogeneity and diversity are hallmarks of GEP-NENs, although they share a common origin from cells of the gut (9) and express neural and endocrine immunohistochemical markers as synaptophysin, neuron-specific enolase, and chromogranin A. Indeed, they differ for biological behavior, presence/absence of a clinical syndrome due to hormone release, malignant potential, and molecular anomalies (8, 10). This variability is evident not only among different sites of origin but also within tumors of the same anatomical site (11, 12).

Initial information about the molecular alterations underlying the development of GEP-NENs came from the study of genetic syndromes associated with the emergence of endocrine neoplasms throughout the patient’s body. In the last 10 years, a rapid increase in data publication has been driven by next-generation sequencing and other high-throughput techniques (microarray expression, miRNA and methylome analysis), especially on pancreatic and small intestinal NETs (13–22).

As a consequence, a large number of genetic and epigenetic alterations have been reported. Recurrent deregulations have been traced back to a reduced number of core pathways. These include DNA damage repair, chromatin remodeling/histone methylation, telomere alteration, phosphoinositide 3-kinase (PI3K)/mammalian target of rapamycin (mTOR) signaling pathway, and cell cycle/proliferation; approved drugs such as sunitinib and everolimus offer possible therapeutic options for the latter (23–25).

Alterations reported also confirmed a radical difference between well-differentiated NETs, including those with a high proliferation index, and NECs. In fact, the diverse morphological features and clinical behavior of these two entities (26) are mirrored by their mutational landscapes: NECs display frequent inactivation of *RB1* and *TP53*, which instead are rare events in NETs (27). These differences are now better reflected by the latest WHO 2017 classification of pancreatic NETs (PanNETs), which is anticipated to be extended also to the other GEP-NETs in the near future (8, 28).

Despite the large amount of generated information, it is still difficult to identify the key nodes (*i.e.*, proteins that interact with many partners and whose alteration can have disruptive consequences) and interactions inside the deregulated pathways that lead to the large heterogeneity featured by these tumors.

One reason for this is the relatively fragmentary nature of the research so far. This is caused by the low incidence of these diseases. Other limitations include the amount of tissue available for research and the need to run integrated analyses for both genetic and epigenetic drivers. As a consequence, there are few really integrated studies to date and they usually include fewer than 100 cases. Moreover, these studies show only partial overlap of cases that could be profiled for DNA, RNA, and methylation/epigenetic analyses (19, 20).

A second reason resides in the paucity of disease models. The attempts to establish patient-derived xenografts have failed to date, whereas available cell lines (including the most frequently used BON-1 and QGP1) and the Rip1Tag2 genetically engineered mouse model are genetically closer to NECs than to well-differentiated NETs. Therefore, for a long time researchers questioned whether they could adequately recapitulate the disease (17, 29–32).

This situation has an impact on both diagnostic and prognostic attempts to subclassify these tumors, as well as on planning personalized therapeutic strategies. Indeed, knowledge of the underlying biology is essential to anticipate possible resistance mechanisms to first-line therapies and to devise alternative plans (33).

This review summarizes present knowledge on genetic and epigenetic alterations implicated in GEP-NEN oncogenesis and progression. Rather than focus on alterations of each single compartment [mutations, copy number variations (CNVs), gene fusions, methylome, miRNAs], the aim is to integrate the available information through analysis of the signaling pathways. This will provide a summary of the key nodes that drive the survival of neoplastic cells while clarifying similarities and differences in these nodes among GEP-NENs of different sites. It will also highlight points that need further investigation to fully understand and anticipate the response of tumors to present and upcoming treatments.

## Basic Histology and Biology of GEP-NENs

**Figure 1.**
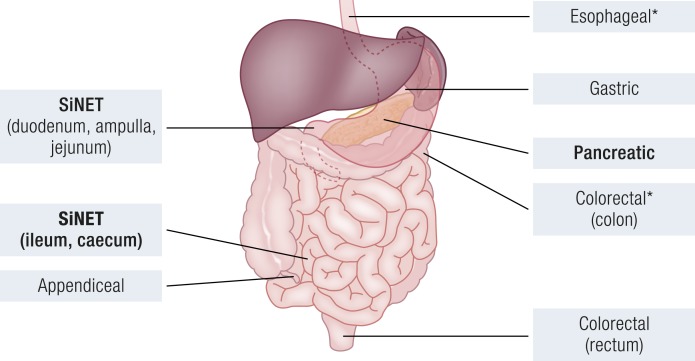
Anatomic location of GEP-NENs. Sites where large-scale genetic and epigenetic data are available are in bold; sites with preponderance of poorly differentiated carcinomas are marked with an asterisk.

A summary of the similarities and differences of GEP-NENs regarding the anatomic region where they develop and grow (Fig. 1) is necessary to understand the implications of their genetic and epigenetic changes. Moreover, although for some organs there has been intensive molecular investigation (*i.e.*, small intestine and pancreas), others lack any integrated data sets. Therefore, it is not clear to what extent the conclusions drawn from tumors of the most studied sites may be extended to tumors from less studied sites.

### Site of origin and main differences

GEP-NENs may arise along all the digestive tract, from the esophagus to the rectum. They are thought to originate from the neuroendocrine cells that form organoid aggregations or are disseminated in the organs (34, 35). Well-differentiated NETs are more prevalent at most sites, with the exception of esophagus and colon, where poorly differentiated NEC is the predominant neoplasm (34).

#### Esophageal NENs

The esophagus is the organ with the lowest incidence of GEP-NENs (<0.1% of all GEP-NENs). They are usually poorly differentiated carcinomas or mixed endocrine/nonendocrine carcinomas in a context of Barrett’s esophagus (36, 37). Most of them are “nonfunctioning,” that is, they grow without causing a clinical hormone-related syndrome and are discovered owing to nonspecific symptoms. Whereas NECs are large, invasive, and have a dismal prognosis, the much rarer NETs tend to be <4 cm and are virtually cured by resection alone (38). There are no substantial genetic data available for these neoplasms in the literature.

#### Gastric NENs

Gastric NENs amount to 5% of all GEP-NENs. Their incidence has been rising in recent years, also due to the growing availability of endoscopy (4, 38). Their 5-year survival is heavily dependent on tumor subtype, though they all arise from hyperproliferating enterochromaffin-like cells of gastric fundus (37, 39).

Type 1 tumors are the most common (80% of cases). They are associated with hypergastrinemia, chronic atrophic gastritis, and achlorhydria and usually appear in the form of multiple nodules of low grade (G1) as measured by mitotic activity (Table 1). They are usually small enough to be removed endoscopically, and therefore they have a good prognosis. Tumor-specific death is 1% and associates to larger size of the tumor (40).

**Table 1.** Evolution of the WHO Classification of GEP-NENs From 2000 to 2017

Molecular Biology*^a^*	WHO 2000*^b^*	WHO 2010*^c^*	WHO 2017 (Pancreas)
Tumor	Well-differentiated endocrine tumor	NET G1	NET G1
	• Benign (small, no invasion, Ki-67 <2%)	• Mitoses per 10 HPF <2	• Mitoses per 10 HPFs <2
	• Uncertain behavior (size >2 cm, or Ki-67 >2%, or angio-neuro invasion)	• Ki-67 ≤2%	• Ki-67 <3%
Tumor		NET G2	NET G2
		• Mitoses per 10 HPFs ≥2, ≤20	• Mitoses per 10 HPFs ≥2, ≤20
		• Ki-67 >2%, ≤20%	• Ki-67 ≥3%, ≤20%
Tumor	Well-differentiated endocrine carcinoma (metastatic)	NEC G3 (poorly differentiated, large or small cell)	NET G3 (well differentiated)
		• Mitoses per 10 HPFs >20	• Mitoses per 10 HPFs >20
		• Ki-67 20%	• Ki-67 >20%
Carcinoma	Poorly differentiated endocrine carcinoma (large or small cell)		NEC G3 (poorly differentiated, large or small cell)
			• Mitoses per 10 HPFs >20
			• Ki-67 >20%

Abbreviation: HPF, high-power field.

^a^ Tumor/Carcinoma: absence/presence of *TP53* and *RB1* recurrent alterations

^b^ Simplified version that merges the WHO 2000 classification of gastrointestinal NETs and the WHO 2004 classification of PanNETs

^c^ From WHO 2010, all NETs are classified as having malignant potential.

Type 2 tumors are quite similar to type 1 as for multicentric presentation and low grade. However, they arise in the context of multiple endocrine neoplasia (MEN) type I (MEN1) genetic syndrome and are associated with the Zollinger–Ellison syndrome by gastrin hypersecretion. They also show a higher metastatic potential compared with type 1 tumors (41, 42).

Type 3 is referred to as “sporadic,” as it develops in the absence of any specific gastric pathology or genetic syndrome, in the context of a nonhyperplastic or nondysplastic mucosa. These tumors constitute ∼23% of gastric NETs. They are larger and more aggressive than the previous two types, with half cases displaying some extent of infiltration and lymph node or distant metastasis (40, 43). Disease-specific death varies from 0% to 26% in the literature, suggesting large heterogeneity in their actual aggressiveness (41, 44).

Type 4 is gastric NEC, which is quite rare, with reported incidence rates ranging from 6% to 16% of all gastric NENs. They are morphologically heterogeneous, with major types being large and small cell carcinoma and the prevalent growth pattern being the solid one (8, 41, 45). Similar to NECs of other sites, they have a poorer prognosis (11 to 82 months) than do their NET counterparts and often coexist with an adenocarcinoma component (46).

Molecular data in the literature are quite sporadic: gastric NECs have been shown to harbor mutation of the *TP53* gene as the most recurrent alteration, affecting from 53% to 100% of cases (47–50). Loss of heterozygosity (LOH) at the *TP53* and *SMAD4* loci and at chromosome 6q was detected as well (49, 51, 52). One whole-exome sequencing study reported that gastric NECs and adenocarcinomas shared frequent mutations of *TP53* and rare mutations of *SYNE1*, but NECs displayed a higher mutation rate than did gastric adenocarcinoma (47). All of these studies, however, included a low number of cases (≤15). Therefore, the only clear information is the frequent alteration of *TP53*, although sporadic mutations in other typical NEC genes (*KRAS* and *RB1*) have been described (47, 53). Regarding NETs, the only whole-exome study to date focused on a family with 5 of 10 siblings affected by gastric type 1 NETs. A germline mutation in the *ATP4A* gene was associated with the disease, but mutations of this gene were not detected in a validation cohort of 14 sporadic type 1 NETs (54). Other studies performed on small cohorts have highlighted LOH at chromosome 11p13, where the *MEN1* locus resides, and hypermethylation at the *CDKN2A* locus as possibly frequent events in gastric NETs (∼50% of studied cases) (49, 51, 52).

#### Small intestine NENs

Small intestine NENs (SiNENs) are the most common NENs of the digestive tract in the Western world (25% to 30% of all GEP-NENs), whereas they are infrequent in Eastern countries and very rare in South Asia (4, 55, 56). The WHO 2010 classification divided them into two subgroups of small intestine NETs: tumors of the duodenum-proximal jejunum (proximal SiNETs) and tumors of the distal jejunum/ileum (distal SiNETs). These subgroups are characterized by different features, although they have been often pooled in epidemiological and molecular studies (4, 8, 15, 19).

Proximal SiNETs account for 5% to 8% of all GEP-NENs, with increasing rates in more recent series. They may be associated to genetic or clinical syndromes but 90% of them are sporadic and nonfunctioning, and the vast majority are located in the duodenum (8, 57). Nonfunctioning tumors (*i.e.*, not associated with a clinical hormonal syndrome) are usually well differentiated and may produce hormones, most frequently gastrin and somatostatin, albeit not in sufficient amounts to elicit a syndrome (34, 38).

Gastrin-producing nonfunctioning and functioning tumors are indistinguishable based on histology and immunophenotype. However, nonfunctioning tumors tend to be small (<2 cm), polypoid, and rarely present metastases (34, 38). Conversely, a large fraction of functioning tumors (gastrinomas) have already metastasized at the time of diagnosis. The metastasis is often larger than the primary tumor, which is even smaller than its nonfunctioning counterpart (58, 59). Nearly one third of these gastrinomas are found in patients with MEN1 genetic syndrome. In that case, they are multicentric, although biological behavior and tendency to metastasis are similar to those of sporadic cases (59–61).

Somatostatin-producing tumors arise in proximity of the ampulla of Vater; they are glandular, larger than gastrin-producing tumors (average size of 2.3 cm), and the presence of metastasis correlates with size and pattern of invasion of the tumor. About 40% of them are detected in the context of neurofibromatosis type I (NF1) genetic syndrome (62); a small number of cases have been reported secondary to mosaic mutations of the hypoxia-inducible factor (HIF) gene *HIF2A* (63, 64).

NECs are rare, usually occur around the ampulla of Vater, and do not express hormones. When they are diagnosed due to jaundice or hemorrhage they often feature lymph node and distant metastasis (65, 66).

Distal SiNETs mainly arise in the terminal part of the ileum. Their average size is 1 to 2 cm; lymph node metastasis correlates with size and is virtually always present when the tumor is >2 cm; liver metastasis is also often present at this point (67). They express serotonin, and its secretion may increase over time due to tumor growth or liver metastasis. This may cause the carcinoid syndrome characterized by chronic diarrhea, flush attacks, bronchial constrictions, and eventually right-sided heart failure. Nonsyndromic cases are usually discovered by chance or because they cause obstruction; in the second case they are often metastatic, as for 95% of syndromic cases (67). Surgery is the first-choice treatment, but it is hampered by the presence of metastases, and the 5-year survival rates drop from 60% to 35% in the presence of liver metastasis (2, 67).

There is no clear association with known genetic syndromes, but familial cases have been observed and about one third of cases present as multicentric tumors, similarly to MEN1-associated gastrinomas. Moreover, 15% to 29% of them coexist with another tumor at the time of diagnosis (34, 68, 69). Individuals from families with more than one first-degree relative affected by a SiNET have a higher risk of developing this disease (70). This is discussed in detail in “Hereditary SiNETs” below.

The genetics of SiNETs remain elusive. They have been recently investigated by multiple groups at the genomic and epigenomic level, providing dissonant information. In particular, the lack of a driver mutation has puzzled the researchers, although the report of somatic mutations scattered across genes of several proproliferative pathways suggested a multigene mechanisms of carcinogenesis (15, 16, 71). Differently from gene mutations, the analysis of gene/chromosomal losses and gains concordantly identified loss of chromosome 18 in ∼50% of cases and gain of chromosomes 4, 5, 7, 14, and 20 occurring in a range from 10% to 30% of cases. Involved genes pointed to the mTOR and TGF*β*/Wnt pathways (15, 16, 71–75). Epigenetic studies have involved mainly methylation analysis, showing an interesting contrast: the DNA of SiNETs is globally hypomethylated, although hypermethylation has been detected on selected tumor suppressor genes, suggesting that they may be involved in tumorigenesis (19, 76–79). Global methylation analyses also allowed clustering of SiNETs in three groups showing association with disease aggressiveness (19, 79). This is discussed in detail in “Genetic and epigenetic alterations of SiNETs” below.

#### Appendiceal NENs

Appendiceal NETs are the second most frequent GEP-NEN according to the latest epidemiological studies (4). They are often incidental findings during appendectomy, and the median age at diagnosis is 38 years, much lower than the other GEP-NENs (8, 80). They are usually small (1 to 2 cm), with a solid insular-like histological pattern, rare mitoses, and low atypia. In most cases cells produce serotonin and are positive for the classical neuroendocrine markers (34). Prognosis is usually extremely favorable, with only 3% patients dying of disease. Tumor size and the pT tumor stage have been indicated as poor prognosis predictors, although the WHO 2010 classification and grading seemed less effective (80, 81). The studies to date, however, rely on a small number of subjects considering the low mortality of the disease. This limits the significance of analyses and suggests caution.

The limited available molecular data focus on goblet cell carcinoids (GCCs), a particular subtype (6% of appendiceal NENs) that is not considered a “pure” carcinoid. Indeed, it expresses mucin and can have a frank carcinomatous component (82). These data have shown the absence of mutation/deregulation only in carcinoma-related genes such as *KRAS*, *BRAF*, *TP53*, *SMAD4*, and mismatch repair genes (82, 83). Only one study compared the profile of appendiceal NETs (three cases) against GCCs with (nine cases) or without (four cases) a carcinoma component. Three hundred seventy-nine cancer-related genes were investigated, and no mutation was reported in appendiceal NETs. One GCC was mutated for *CDH1*, whereas GCCs with a carcinoma component displayed mutations in *ARID1A*, *RHOA*, *KDM6A*, and *SOX9* (84).

#### Colorectal NENs

Colon NENs are infrequent (0.2 new cases per 100,000 per year) (4). NETs are usually small, nonsyndromic, and identified during screening colonoscopies. Similarly to the esophagus, most cases are poorly differentiated NECs that are >2 cm and cause symptoms due to aggressive growth (34, 38). Colon NETs have a 5-year survival of 62%, and prognosis largely depends on the presence of invasion and metastasis. NECs are very aggressive and often metastatic at the time of diagnosis; they have an even poorer prognosis than do adenocarcinomas (34, 85, 86). From the molecular point of view, colon NECs are similar to colorectal adenocarcinoma, with recurrent mutation of *APC*, *KRAS*, *BRAF*, and *TP53;* the occurrence of microsatellite instability has been described as well (53, 87). Nonetheless, they also show features of NECs from other sites such as decreased expression of Rb and overexpression of p16 and Bcl-2 (53, 87). When they are found as mixed adenoneuroendocrine carcinomas, the two components of the tumor share most driver mutations but differ for less prevalent alterations. This suggests a common origin with early separation of glandular and neuroendocrine components during oncogenesis (88, 89).

Rectal NETs are almost invariably identified during screening colonoscopy, and their incidence has risen to 1 in 100,000 per year in the United States, whereas worldwide it is ∼0.2 (4). The high rates in the United States are partly due to the higher incidence of this disease among people of African descent (2). Their median size is 0.6 to 1 cm, and in this case the tumor can be resected endoscopically. Prognosis is generally fair: 88% of patients are alive at 5 years and 3% of cases display lymph node metastasis. Tumors of larger size (˃2 cm) and/or with muscular invasion are proportionally more likely (50% to 80%) to present lymph node involvement and to have a poorer outcome (34, 90, 91). A recent molecular analysis has reported absence of microsatellite instability and of mutations in *KRAS*, *NRAS*, *BRAF*, and *PIK3CA.* The same study showed mutual association between lymph node invasion, CpG island methylation, and miR-885-5p expression (92). Finally, whole-genome sequencing on six liver metastases from the same patient showed that 11 of 18 somatic mutations were identified in all samples, including known tumor-related genes *HSPG2*, *SERPINF2* (extracellular matrix remodeling), and *SMARCA1* (chromatin remodeling) (93).

#### Pancreatic NENs

Pancreatic NENs (PanNENs) account for 15% to 20% of GEP-NENs (4). Although most (60% to 85%) are nonfunctioning, functioning ones display a broad range of hormone-related syndrome types, according to the different hormones produced by cells of the pancreatic islets. The most frequent functioning PanNETs include insulinoma, gastrinoma, glucagonoma, VIPoma, and somatostatinoma (8, 34, 94, 95). Nonfunctioning tumors were usually discovered at a more advanced stage, when the growing mass caused symptoms such as jaundice. However, thanks to the improvement in radiological imaging, tumor size at diagnosis has progressively decreased over the years (34).

The vast majority (92.5%) of PanNENs are NETs with a highly variable prognosis; 5-year survival ranges from 29% to 70% and largely depends on tumor grade and stage. Pancreatic NECs account for 7.5% of PanNENs, display recurrent *TP53* and *RB1* mutations, and have a dismal outcome, similar to other NECs of the gastroenteric tract (27, 40, 96, 97).

Whereas PanNETs are usually monocentric, the presence of multiple neoplastic foci may indicate a genetic syndrome. About 10% of PanNETs occur in the context of MEN1, von Hippel–Lindau (VHL), tuberous sclerosis (TS), or NF1 syndromes. These genetic syndromes are caused by germline mutations in genes that gave the first insight into the molecular biology of these tumors (detailed in “Genetic syndromes” below). In particular, *MEN1* and *TSC1/2* involvement were confirmed by recent studies, which also found novel recurrent targets for mutation (14, 98–101). These data were further expanded by whole-genome and epigenetic analyses that added important information on the alteration of core pathways in PanNETs (20, 77, 102). Details are further discussed in “Genetic and epigenetic alterations of PanNETs” below.

### NETs vs NECs (WHO 2010–2017)

NENs of all sites have been classified by the diverse editions of the WHO “blue books” across the years (Table 1). Histological features and, successively, tumor grading based on proliferation measure (mitotic count and/or Ki-67 index) were progressively integrated to this end.

The classification published in 2000 and 2004 categorized GEP-NENs into three groups: well-differentiated endocrine tumor, well-differentiated endocrine carcinoma, and poorly differentiated endocrine carcinoma. The difference between well-differentiated tumors and carcinomas was determined by the presence of invasion or metastasis. The poorly differentiated carcinomas were defined by loss of tissue architecture and severe cell atypia. Whereas well-differentiated endocrine tumors may vary widely, histological features of poorly differentiated endocrine carcinomas are similar across different organs. They were subclassified into large-cell and small-cell carcinoma based on cellular morphology.

*“Studies on mixed neuroendocrine/nonneuroendocrine carcinomas showed that driver alterations were shared…”*

Tumor grading of GEP-NENs was introduced by the WHO 2010 classification endorsing the European Neuroendocrine Tumor Society consensus proposal of a three-tier system (103, 104). A mitotic count of <2 per 2 mm^2^ and/or a Ki-67 index <3% identifies NET G1, a mitotic count of 2 to 20 per 2 mm^2^ and/or a Ki-67 index of 3% to 20% identifies NET G2, and a mitotic count of >20 per 2 mm^2^ and/or a Ki-67 index >20% leads to an NEC definition (8). However, the WHO 2010 classification defined all G3 neoplasms as NECs and equalized them to poorly differentiated tumors; it was assumed that no well-differentiated endocrine tumors with a mitotic count/Ki-67 index >20% could exist (105).

This classification of G3 tumors has been challenged by later publications and molecular studies showing that well-differentiated tumors may have a proliferation index in the G3 range. These neoplasms differ from poorly differentiated NECs for prognosis, mitotic counts, and Ki-67 levels. Moreover, they substantially lack mutation of *TP53* and *RB1*, which instead are pivotal drivers in poorly differentiated NECs of any anatomical origin (20, 26, 40, 106–108).

The mutation of *TP53* or the nuclear accumulation of its gene product p53 has been repeatedly detected in poorly differentiated GEP-NECs, with frequency ranges of 20% to 73% for mutation (27, 47, 50, 87, 109) and 65% to 100% for nuclear accumulation (48, 65, 87, 110–112). Similarly, *RB1* inactivating mutations and concomitant loss of Rb immunolabeling were reported in 71% poorly differentiated pancreatic NECs (27). Lack of Rb immunostaining is a recurrent event also in gastric, colorectal, and ampullary NECs, with frequencies ranging from 44% to 86% across different studies (48, 65, 87, 113). Rb is a key negative regulator of the cell cycle: it inhibits progression from G_1_ to S phase, cooperating with p16 and other proteins (114). Interestingly, a subset of NECs displayed loss of p16 immunostaining (20% to 44%), alone or in combination with Rb loss (27, 48, 65, 87). Parallel studies also showed frequent methylation (10% to 60%) at the promoter of *RASSF1A*, another cell cycle repressor acting via the Rb-mediated checkpoint (115–118). All of these data remark the importance and the high level of cell cycle deregulation in NEC tumorigenesis.

*KRAS* mutations have been described in gastric, pancreatic, and colorectal NECs (frequency range, 8% to 60%; median, 30%) (27, 53, 86–89, 109, 113), whereas *BRAF* mutations (frequency range, 13% to 59%; median, 17%) were found only in colorectal NECs (53, 86, 109, 118, 119).

Studies on mixed neuroendocrine/nonneuroendocrine carcinomas showed that driver alterations were shared by both components. The most prevalent genetic events included mutations/loss of *TP53* and *RB1* and mutation of *KRAS*. Colorectal cases also displayed *APC* mutation (50, 87–89, 96, 117, 120).

Although lacking the genetic alterations that characterize poorly differentiated NECs, G3 NETs bear driver alterations that are a hallmark of G1/G2 NETs. In particular, mutation or protein loss of MEN1, DAXX, and ATRX has been detected at high frequency in G3 PanNETs (31% to 44%, 9% to 25%, and 18% to 36%, respectively). These alterations were even suggested as a guide to distinguish G3 PanNETs from NECs when the histological examination is challenging (121, 122). Recent publications have started characterizing G3 NETs from the clinicopathological point of view. These studies have estimated that G3 NETs amount to one third of all G3 NENs and are located preferentially in the pancreas, stomach, and colon/rectum. They have a median Ki-67 (35%), which is lower than that of NECs (70%), and their median survival is four times longer than for NECs but shorter than for G2 NETs (26, 105, 106, 123, 124). Beyond the molecular similarities between G1/G2 and G3 NETs, some differences are expected. Comparative genomic studies are still missing and will be critical to understand these differences (125).

**Figure 2.**
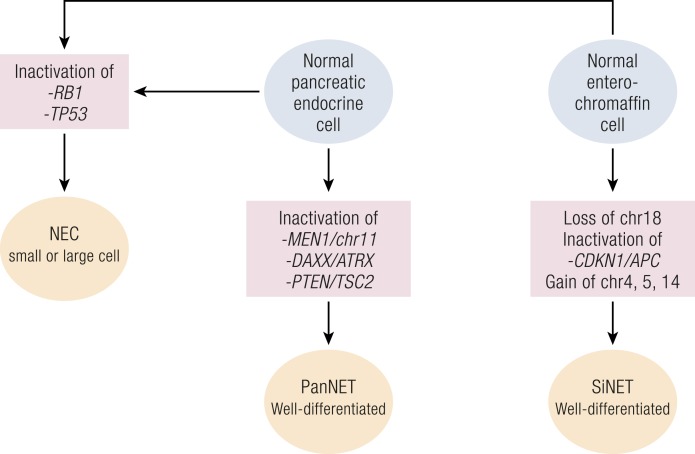
Different molecular genesis of NETs and NECs. Main histological and molecular characteristics of NECs are similar throughout the body, and their aggressiveness is comparable to that of adenocarcinomas, whereas NETs are more indolent.

The above-reported data prompted a new model for GEP-NENs tumorigenesis (Fig. 2). In this model, poorly differentiated NECs and well-differentiated (including G3) NETs develop from a normal neuroendocrine progenitor through different routes. These foresee the alteration of *TP53* and *RB1* for all poorly differentiated NECs, as well as specific alterations for well-differentiated PanNETs and SiNETs (126).

The heterogeneity among WHO 2010 G3 NECs has been ratified for PanNETs with the new WHO 2017 classification of tumors of neuroendocrine organs. G3 NENs are therein divided into G3 NETs and G3 NECs according to their histological differentiation features (10). Moreover, several groups have proposed the use of *RB1*/Rb and *TP53*/p53 molecular alterations to aid the classifications of those ambiguous cases where histology alone cannot reach a definitive diagnosis (105, 122, 126, 127). This is of particular value for NECs that are usually discovered at an advanced stage, when diagnosis often relies on limited assays performed on limited amounts of biopsy tissue.

Considering the profound differences in histological, genetic, and biological features of these two categories, for the rest of the review we focus on the alterations of well-differentiated NETs, starting with the knowledge accumulated thanks to genetic syndromes.

### Genetic syndromes

Similar to other types of cancer, the first clues about GEP-NEN molecular tumorigenesis came from hereditary syndromes associated with the development of NENs. GEP-NENs arising in these contexts are ∼10% of all NENs (128). Knowledge of a hereditary pattern in the pregenomic era allowed mapping and study of the genes responsible for susceptibility to these diseases. These were then investigated also in sporadic tumors, permitting determination of their impact in a more general framework (Table 2).

**Table 2.** Genetic Syndromes Associated With GEP-NETs

Genetic Syndrome	Germline Mutation [Gene (Protein)]	Corresponding Somatic Tumor Site*^a^*
MEN1	MEN1 (menin)	Pancreas
MEN4	*CDKN1B* (p27)	Pancreas; small intestine
VHL	*VHL* (pVHL)	Pancreas
NF1	*NF1* (neurofibromin)	Ampulla of Vater
TS	*TSC1* (hamartin)	Pancreas
	*TSC2* (tuberin)	
Hereditary SiNET	*IPMK* (IPMK)	Pancreas; small intestine*^b^*
	*MUTYH* (hMYH)	
	*OGG1* (AP lyase)	

^*a*^ For each syndrome and germline mutated gene, sites of the corresponding sporadic tumors with alteration of the same gene are displayed.

^*b*^ Only *MUTYH* mutations have been reported in sporadic tumors.

Most of these syndromes and genes have been related to PanNETs or gastric NETs and include the MEN, VHL, NF1, and TS syndromes (129–132). The field of familial SiNETs, alternatively, is relatively new and the causal link between the disease and several reported genes still needs to be validated (133, 134).

#### MEN type I

MEN1 is an autosomal dominant disease, determining high risk of developing parathyroid (95%), pancreatic (60%), and pituitary (30%) endocrine tumors (135, 136). It is caused by germline-inactivating mutations in the *MEN1* gene (137, 138) coupled with somatic loss of the remaining normal allele (139). As mentioned above, PanNETs in this context are usually multicentric even when at the microadenoma stage, supporting the idea of an early involvement of *MEN1* in the neoplastic process (140).

The gene product of *MEN1*, menin, is a ubiquitously expressed protein mainly residing in the nucleus. There, it has been shown to interact with a high number of proteins involved in histone methylation, cell signaling, or transcriptional activation. MEN1 activity mediates a large number of homeostatic activities, including (i) increased histone methylation and expression of the *CDKN2C/CDKN1B* cell cycle inhibitors, by associating with a complex containing histone methylases KMT2A/2D (also known as MLL1/2) (141); (ii) inhibition of the PI3K/mTOR signaling pathway by reducing translocation of Akt1 from cytoplasm to plasma membrane (142); and (iii) activation of genes of the homologous recombination (HR) DNA repair machinery (*BRCA1* and *RAD51*) in response to DNA double-strand breaks (143, 144).

*MEN1* gene alteration has been also reported in a large fraction of sporadic NETs; it is considered one of the pivotal genes in NET biology, although its full spectrum of action has not been completely elucidated (14, 20, 98, 145–147).

#### MEN type IV

MEN type IV (MEN4) has been described relatively recently as an addition to the MEN spectrum (148). The onset of the disease is in the third decade of life and is characterized by the appearance of parathyroid and pituitary tumors. These are sometimes accompanied by gastric, pancreatic, and bronchial NETs or gastrinomas. Initially discovered in rats lacking mutations in *Men1* and *Ret* (the gene responsible for MEN2), it was named MENX because of its unknown driver gene. It was subsequently linked to inactivating germline mutations of the *Cdkn1b* gene, and this association was later confirmed in humans (149). It was also shown that heterozygous mutation of *CKDN1B* was associated to lack of its gene product—the p27 protein—in the affected tissues due to gene haploinsufficiency, that is, the inability of a gene to preserve its normal function when even only one copy of the gene is deleted or inactivated (149–154). These data, and the report that *MEN1* mutation leads to loss of p27 expression (155), support the involvement of p27 in early phases of NET development.

#### von Hippel**–**Lindau syndrome

The autosomal dominant von Hippel**–**Lindau syndrome is caused by inactivating germline mutations in the *VHL* gene. Its clinical features encompass the onset of NENs, including PanNETs in 8% to 17% of patients (156). In particular, mutations in exon 3 of the gene associate with PanNETs (157). The gene product (pVHL) is a negative regulator of the HIFs, a set of transcription factors activated by the PI3K/mTOR pathway and controlled by pVHL via ubiquitination. Loss of pVHL function results in increased expression of vascular endothelial growth factor receptor (VEGFR) and platelet-derived growth factor receptor (PDGFR), two receptor tyrosine kinases. Upon binding of their ligands, VEGFR and PDGFR may activate signaling in a feed-forward loop with the PI3K/mTOR pathway itself (158). *VHL* mutation is rare in sporadic PanNETs, but its inactivation by gene deletion (18%) or promoter hypermethylation (6%) leads to similar effects (159).

#### Neurofibromatosis type I

Neurofibromatosis type I, a genetic disease, has a relatively high birth incidence (1 in 2500); its clinical features include cutaneous neurofibromas, skinfold freckling, café au lait spots, iris Lisch nodules, and nervous tumors or NETs (160). About 10% of NF1 patients develop a GEP-NET, usually a periampullary or duodenal somatostatinoma. As a consequence, 40% of these rare tumors are found in association with *NF1* germline alterations (161–163). Patients suffering from NF1 inherit germline-inactivating mutations of the *NF1* gene that cause deep deregulation of both rat sarcoma (Ras)/mitogen-activated protein kinases (MAPK) and PI3K/mTOR signal transduction networks. Indeed, *NF1* gene product (neurofibromin) is a negative modulator of both pathways: it converts Ras-GTP to its inactive form, Ras-GDP, and indirectly inhibits Akt-mediated TSC2 phosphorylation and inactivation (164, 165).

#### Tuberous sclerosis

TS features the direct deregulation of the PI3K/mTOR signal transduction network, which was indirectly involved in the previous genetic syndromes. In fact, the disease is caused by inactivating mutations in either of two genes, *TSC1* and *TSC2* (166, 167). Their products, hamartin and tuberin, act as a complex to inhibit mTOR signaling. In healthy cells, they are negatively regulated by Akt1. Under appropriate stimuli, Akt phosphorylates tuberin, triggering Ras and PI3K signaling (164, 165). Loss of function of either *TSC1* or *TSC2* therefore causes a hyperactivation of the mTORC1 complex, which is no longer controlled by the TSC complex. This causes the onset of clinical symptoms, usually including facial angiofibromas and widespread hamartomas throughout the body (168). GEP-NETs rarely develop as a consequence of this syndrome (169), but recent works reported downregulation and mutation of *TSC1*/*TSC2* in sporadic PanNETs [discussed in detail in “Genetic and epigenetic alterations of PanNETs” below (14, 99)].

#### Hereditary SiNET

The definition of hereditary SiNET, also known as familial SiNET, is relatively recent. Existence of a familial form of SiNET was initially hypothesized based on risk epidemiological reports. The risk of developing SiNET was found to be 3.6-fold higher when at least one first-degree relative was affected, in the absence of known genetic syndromes (70). Consistent with this observation, active surveillance allowed detection of tumors at an earlier stage in families with hereditary SiNET. The inheritance pattern so far seems to follow an autosomal dominant pattern, according to the largest pedigree study to date, which included nine families (73). The same study showed loss of chromosome 18q, similar to sporadic SiNETs but without specific mutations. Several reports confirm that both familial (38%) and sporadic (43% to 68%) SiNETs may display chromosome 18 copy loss, suggesting a similar pathogenesis in at least a fraction of them (15, 16, 19, 72, 73, 170).

A subsequent study confirmed increased risk of SiNET in families with two or more affected members and identified a mutation of the *IPMK* gene in one of the families by genomic profiling. This mutation leads to p53 inhibition and Akt activation, potentially supporting tumorigenesis. However, it was not found in other families to date (134). Another recent study performed a large genomic profiling in 15 families and identified mutations in two genes, *MUTYH* and *OGG1* (133), with homologous function: both are implicated in DNA base excision repair and were previously reported to be involved in neoplastic diseases (171, 172). The same study also showed that the frequency of these mutations was increased in 215 sporadic SiNETs (133), confirming the relevance of altered base excision repair in SiNET tumorigenesis. Moreover, a concomitant study on PanNETs demonstrated the presence of germline *MUTYH* mutations and defective DNA repair also in this tumor type. This suggested that a fraction of the so-called sporadic cases may hide a hereditary nature (20).

#### Lessons from GEP-NET hereditary syndromes

Taken together, data derived from hereditary syndromes and investigation of the affected genes in sporadic GEP-NETs clearly call for an involvement of two main pathways: cyclin-dependent cell cycle regulation (MEN1, MEN4) and the PI3K/mTOR pathway (MEN1, VHL, NF1, TS).

## An Updated Landscape of the Molecular Alterations of GEP-NETs

Although genetic syndromes provided the first clues on the development of GEP-NENs, research on sporadic tumors was limited for a relatively long time by lack of tissue samples. The main cause was the low incidence of these neoplasms, contrasting with the need for abundant tissue samples to be used for cytogenetics, conventional Sanger sequencing, nucleic acid hybridization techniques, or microsatellite-based LOH analysis.

Microarray-based comparative genomic hybridization (CGH) allowed for a first large-scale comparison of GEP-NET chromosomal alterations. The discovery of different patterns of losses and gains between PanNETs (frequent losses at chromosome 1, 3, 6, and 11 and gains at chromosomes 7, 9, 17, and 20) and SiNETs (loss of chromosome 18 in 75% of cases, gains at chromosomes 4, 5, 14, and 20) prompted a first discussion on the possible different natures of PanNETs and SiNETs (72, 73, 170, 173, 174).

The main problem with CGH analysis was the difficulty to link chromosomal copy alterations to specific genes whose loss or gain may drive the neoplastic process. This barrier began to fade with the advent of next-generation sequencing techniques. These new technologies offered the possibility of producing single-nucleotide resolution data for DNA, RNA, methylation, and chromatin analyses from limited amounts of nucleic acid (0.1 to 10 µg), with a turnaround time of a few days (175, 176). Thanks to next-generation sequencing, large amounts of information on GEP-NET molecular changes have been produced in the last 10 years.

### Genetic and epigenetic alterations of PanNETs

#### Genetic alterations in PanNETs

Most of the sparse literature about PanNET mutations and chromosomal alterations has been clarified by two large-scale studies. These included a whole-exome study by Jiao *et al.* (14) in 2011and a whole-genome/RNA sequencing study by Scarpa *et al.* (20) in 2017.

The two studies mainly included nonfunctioning G1/G2 PanNETs and confirmed previous low-throughput studies regarding frequent mutation of *MEN1* in ∼40% of cases. They also confirmed inactivation of *TSC1* and *TSC2* (∼6% of cases) and deregulation of the PI3K/mTOR pathway in PanNETs, which was initially suggested by expression profiling studies (99). This concept was further strengthened by another finding, that is, the identification of recurrent (7% of cases) inactivating mutations of *PTEN*, a gene involved in PI3K inhibition. Despite that most analyzed cases were locally advanced or metastatic, low mutational rates were reported. Furthermore, genes commonly affected in pancreatic ductal adenocarcinoma or NEC (*i.e.*, *TP53* and *RB1*) were never or rarely found to be altered.

A second important finding reported by Jiao *et al.* (14) is the identification of two novel recurrently mutated genes: *DAXX* (25%) and *ATRX* (17%). Their mutation was mutually exclusive and associated with loss of protein expression, in keeping with their reported cooperation as a complex to deposit histone H3.3 at telomeres (177). Moreover, their mutation in PanNETs was associated with a phenomenon called alternative lengthening of telomeres (ALT) (100).

When ALT is triggered, the progressive shortening of telomeres that naturally occurs is reversed without a direct activation of the telomerase. Instead, nuclear bodies containing a large number of telomeric DNA repeats accumulate in neoplastic cells. This happens through a mechanisms that is mediated by HR but is repressed in the presence of the DAXX/ATRX complex (178).

**Figure 3.**
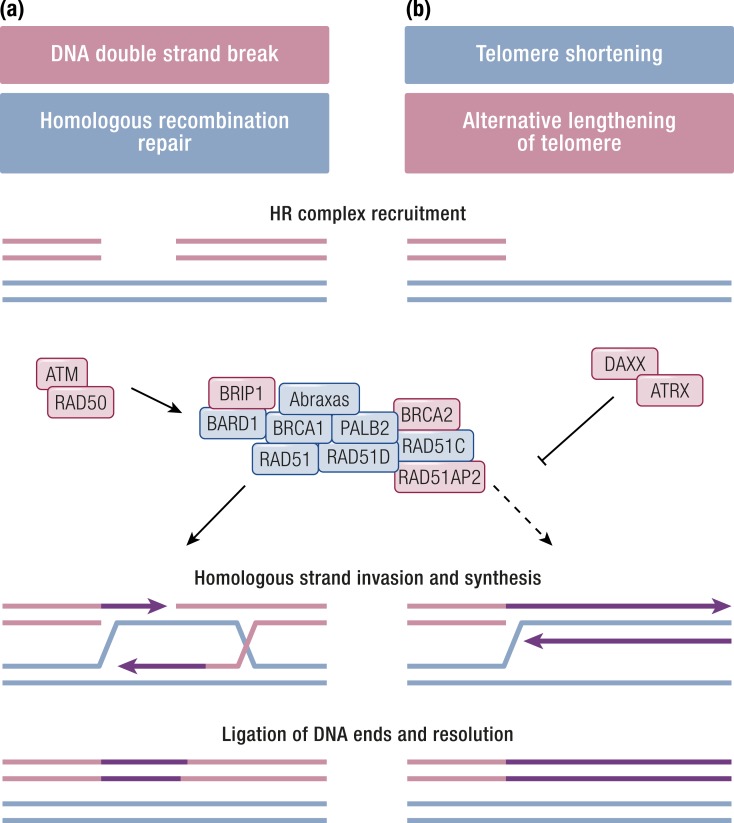
HR repair of DNA double-strand breaks and its deregulation in ALT. (a) In normal conditions, DNA double-strand breaks trigger the HR complex via ATM and RAD50. The complex repairs broken DNA strands using the complementary strand of the intact sister chromosome as a template. (b) This is normally inhibited on shortening telomeres by the DAXX/ATRX complex. In a fraction of NETs, loss of DAXX/ATRX function causes the HR complex to attempt “repair” of telomeres, resulting in ALT. Proteins whose gene has been reported as altered in neuroendocrine tumors are shaded in red.

The HR DNA repair complex is a molecular machinery that repairs double-strand breaks in a chromosome by using the homologous region in the sister chromosome. A number of genes contribute to the repair machinery, including *ATM*, *BRCA1*, *BRCA2*, *RAD50*, and *PALB2*, and their inactivation has been initially studied in the context of hereditary breast and ovarian cancer (179). In PanNETs featuring ALT, however, the loss of DAXX/ATRX causes the HR complex to attempt the “repair” of shortened telomeres, which results in the above-described phenotype (Fig. 3) (178).

The correlation between DAXX/ATRX expression loss and ALT was confirmed by independent studies. According to these reports, a small number (6%) of cases showed ALT in the absence of DAXX/ATRX loss, which suggested the ancillary interplay of other proteins (180, 181).

ALT and DAXX/ATRX loss have been correlated with higher tumor stage and grade and are therefore considered a late event in PanNET tumorigenesis. Probably due to this association, data regarding their value as independent predictors of disease recurrence are conflicting (180, 181). A partial explanation of these conflicting data may come from a recently discovered interaction between DAXX and PTEN. Indeed, tumor growth is inhibited when DAXX expression is blocked in *PTEN*-deficient glioma cells, a finding that warrants experiments in PanNETs as well (182).

*“Loss of function of either* TSC1 *or* TSC2 *therefore causes a hyperactivation of the* mTORC1 *complex.”*

These data were further expanded with the successive whole-genome analysis of 98 PanNETs (20). *DAXX/ATRX* mutually exclusive alterations were detected in 33 cases with or without *MEN1* mutation/loss, showing an independence of the two features. Moreover, an integrated analysis showed that ALT, mutation/loss of DAXX or ATRX, and a pattern of recurrent chromosome losses were associated with one third of cases (20). Conversely, cases with shorter telomeres and wild-type *DAXX* or *ATRX* were enriched in genomic rearrangement events such as chromothripsis and/or *EWSR1* gene fusions. These gene fusions are known as recurrent alterations in Ewing sarcoma, and the fusion transcripts are under the control of the PI3K/mTOR pathway (183), but their discovery in PanNETs is a novel finding (20).

Other rearrangements and somatic mutations inactivated genes belonging to cell cycle checkpoint (*CDKN2A*, *CDKN1A*, *CDKN1C*), SWI/SNF chromatin remodeling (*ARID2*, *SMARCA4*), histone methylases (*SETD2*, *KMT2C*, also known as *MLL3*), or suppressors of the mTOR pathway (*PTEN*, *TSC1/2*, *DEPDC5*). Low-frequency mutations also affected several members of the MAPK signaling pathway (20). This finding was recently confirmed by Vandamme *et al.* (184), who reported similar mutations in *MAP4K2* and *MAPKBP1* (three cases each) by targeted deep sequencing of 38 PanNETs.

Whole-genome analysis also allowed the definition of five mutational signatures, of which two were particularly interesting: a novel signature associated with the biallelic inactivation of *MUTYH* due to germline mutation and somatic LOH in 5% of cases, and a BRCA signature due to germline mutation of *BRCA2* and somatic LOH in one case. Both genes contribute to DNA repair. *MUTYH* is involved in base excision repair, and its germline inactivation was initially associated to hereditary polyposis (185). *BRCA2* is part of the HR double-strand break repair machinery (Fig. 3), and its germline mutation has been associated to familial breast and ovarian carcinoma (179). This prompted a further search for germline variations as possible drivers of PanNETs, which resulted in the identification of *CHEK2* germline mutations coupled with LOH in 4% of cases. *CHEK2* is activated by ATM upon DNA double-strand breaks and regulates the cell cycle by interacting with both BRCA1 and p53. Its germline inactivation was associated with Li–Fraumeni syndrome and familial breast cancer (179). Of note, other germline mutations were detected despite the 98 cases that were apparently sporadic on a clinical ground. These included six mutations in *MEN1*, one in *VHL*, and one in *CDKN1B* (MEN4), all coupled to somatic LOH. The above findings suggest that a higher than expected proportion of PanNETs may arise due to germline mutations. Interestingly, a large fraction of cases harboring *MUTYH* or *CHEK2* mutations were also mutated for *DAXX*/*ATRX* but not for *MEN1.* This observation suggests an *MEN1*-independent alternative oncogenesis in those PanNETs, while confirming that telomere-affecting mutations intervene at a later stage.

Besides *BRCA2* and *CHEK2*, other sparse mutations were found in ancillary genes of HR DNA repair, such as *RAD50*, *RAD51AP2*, *BRIP1*, and the previously reported *ATM* (3% to 5%) (186). Three genes of mismatch repair (*MSH3*, *MSH4*, and *MSH6*) were also involved, although the impact of these mutations was not defined.

**Figure 4.**
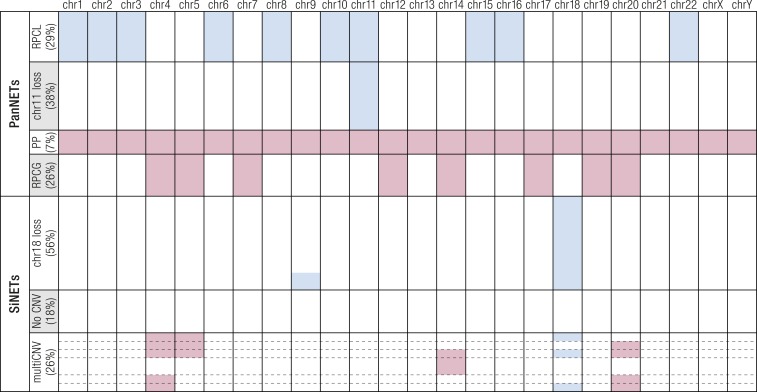
Subtypes of PanNETs and SiNETs according to chromosomal alterations. Four subgroups have been identified in PanNETs and three in SiNETs. Copy gains are shown in red, losses in blue. In each subgroup of tumors, cases with an identical CNV pattern are represented by individual rows, and the height of the row is proportional to the fraction of cases harboring that CNV pattern. chr, chromosome; multiCNV, multiple CNV; PP, polyploid; RPCG, recurrent pattern of chromosomal gains.

Chromosomal CNVs in this study were consistent with previous reports describing recurrent loss at chromosomes 1, 3, 6, 10q, and 11 and gain at chromosomes 4, 5, 7, 12q, 14, 17, 19, and 20 (170, 173, 174). Additionally, clustering of these CNVs showed four distinct groups, revealing that most losses aggregate in a group called “recurrent pattern of chromosomal loss” (RPCL; recurrent loss at chromosomes 1, 2, 3, 6, 8, 10, 11, 15, 16, and 22) or in a second group characterized by loss of chromosome 11 where *MEN1* resides (Fig. 4). The remaining two groups were characterized by gains due to either polyploidy (group 3) or a pattern of chromosomal gains (group 4), which were complementary to losses of the RPCL group. This latter group featured the chromosomal gains previously described in the literature (170, 173, 174) and associated with higher risk of metastasis (187).

RNA sequencing was performed on 30 cases, and clustering of differentially expressed genes revealed the presence of three subgroups (20), and one of them had features overlapping with a previously defined metastasis-prone subgroup (17). Network analysis of transcripts characterizing this subgroup showed an overexpression of genes under the control of the HIF1/2 factors and a concomitant deregulation of glucose metabolism, as predicted by current literature on the role of HIFs in cancer (188). In both works, tumor subgroups and mutations in *MEN1*, *DAXX*/*ATRX*, or the mTOR pathway genes did not associate. Therefore, it is still unclear whether this consistent “HIF signature” is at least partly dependent on genetic determinants or just an adaptation to hypoxic conditions during tumor growth.

Another open question concerns one of the mutational signatures, which has been found in PanNETs and in many other tumors. It has been called “signature 5” because its etiology is unknown to date. However, it has been recently reported that this signature is dominant in *Fhit* knockout mice (189) and in cancer samples of The Cancer Gene Atlas that display *FHIT* gene deletion (190), suggesting a direction for future research on this topic.

Although functioning and nonfunctioning PanNETs share several molecular features, transcriptomics showed that one subtype of functioning tumors, namely insulinomas, is set apart in terms of gene expression (99). This was further confirmed by four whole-exome sequencing studies of sporadic insulinomas that found only rare (2%) MEN1 somatic mutations, but also a frequent hotspot (p.T372R) somatic mutation in the Yin Yang 1 gene. Mutation prevalence ranged from 10% to 30% of cases, with the higher proportion found in Asian patients (191–194). A Sanger sequencing study from India did not find this hotspot Yin Yang 1 mutation in 17 insulinomas (195), whereas a similar study on 23 US patients found two hotspot mutations in 23 (8%) insulinomas (196). This mutation was associated with a late onset of the tumor and was reported to affect YY protein transcriptional activity. Because *YY* is a target of mTORC1, inhibitors such as everolimus have been suggested as a potential therapeutic option (191). However, the exact role of this heterozygous hotspot mutation in the pathogenesis of insulinomas needs further clarification (196). Another gene was recently associated with the onset of insulinomas, namely the V-Maf avian musculoaponeurotic fibrosarcoma oncogene homolog A (*MAFA*) (197). *MAFA* encodes for a transcription factor that is essential for *β*-cell function (198). Its germline alteration has been reported to associate with both insulinomas (preferentially in females) and diabetes (preferentially in males), an observation that needs to be verified (197). Although *MEN1* alterations are rare in insulinomas, an integrative analysis of whole-exome and RNA sequencing data showed that deregulation of genes encoding proteins functionally related to MEN1 play a major role in this tumor type (194). In fact, most cases displayed mutations, CNVs, and/or deregulation in epigenetic modifier genes of the polycomb and trithorax families, reflecting the major role of chromatin remodeling complexes in insulinomas (194).

The most recent works showcase a first integration of genomic and transcriptomic data on PanNETs; this also translates into better knowledge of the epigenetic landscape through the discovery of alterations affecting chromatin remodeling genes. Alternatively, data about DNA methylation and miRNA are more scattered and not flanked by in-depth genomics analyses. Despite this, knowledge has accumulated in this field and contributes to the overall pattern drawn by the main genomic findings.

#### Epigenetic alterations in PanNETs

Methylation of DNA, especially at CpG islands, is a well-known way to regulate gene expression in normal and cancer cells. It may occur in regions proximal to the promoter where an increase in methylation is linked to repression of transcription (199), or in the body of genes where it generally is associated with an increase in expression of the gene product (200).

DNA *de novo* methylation is performed by the DNA methylases DNMT3a and DNMT3b, whereas it is maintained by DNMT1 (201). Hypermethylation seems to affect only gene expression, whereas demethylation in cancer has also been linked to reactivation of mobile elements and chromosomal instability (199). Most studies on PanNETs followed a strategy based on the evaluation of few candidate genes, owing to the inherent technical complexity of DNA methylation analyses.

*RASSF1* has been one of the most studied tumor suppressor genes in this context because its locus was cloned from a region in chromosome 3p21 that is frequently lost in lung cancer. This locus has two promoters, both containing CpG islands, encoding eight isoforms (RASSF1A to RASSF1H). However, only two of them have been extensively characterized: RASSF1A from promoter/CpG island A, and RASSF1C from promoter/CpG island C (202). The promoter for RASSF1A was repeatedly reported as one of the most frequently hypermethylated genes in PanNETs (75% to 83%). Methylation frequency increased in metastatic cases, with an inverse correlation between the degree of methylation and expression of the RASSF1A transcript (116, 203–206). Conversely, RASSF1C was found to be overexpressed in PanNETs, but this was not associated to promoter methylation, which was never detected (77). This antithetic behavior of the two isoforms has been detected in other tumors such as esophageal squamous cell carcinoma (207). It is also consistent with functional data showing that RASSF1A promotes cell cycle arrest and apoptosis by interaction with a large number of proteins, including Ras, c-Jun N-terminal kinase (JNK), APC, and CNK1. Alternatively, RASSF1C promotes the Wnt pathway by inhibiting *β*-catenin degradation and activating JNK. Moreover, it has been shown to be retained by DAXX in the nucleus and released upon DNA damage which triggers DAXX degradation (202, 208, 209).

Another gene that has been reported as hypermethylated in 93% of PanNETs is hypermethylated in cancer (*HIC*)*1*, a downstream effector of *TP53* that is also proximal to the latter, on chromosome 17p13.3. This gene is frequently repressed in a number of cancers, including gastric, breast, hepatocellular, and colorectal carcinoma, either by *TP53* loss of function or by HIC-1 promoter methylation. Its expression correlates with better prognosis and, when restored *in vitro* by demethylating agents, it leads to cell cycle arrest in *TP53* mutant cell lines (206, 210).

The Wnt pathway may be altered in PanNETs also through the *APC* gene, which is responsible for *β*-catenin degradation. Its loss has been associated with increased proliferation and defects in chromosome segregation (211), possibly contributing to chromosomal instability, which is a recurrent feature of PanNETs. Hypermethylation of its promoter has been detected in 21% to 48% of PanNETs (204, 206) as well as loss of expression in 59% (206).

Two other genes have been reported to be hypermethylated in 20% to 50% of PanNETs, namely *CDKN2A* and *MGMT*. The first encodes p16, a tumor suppressor that is frequently inactivated in cancer by copy loss, hypermethylation, or mutation (212). Similar to *RB1* and other members of the *CDKN* family, it is a negative regulator of the cell cycle, and its loss of function leads to proliferation and escape from cell senescence. Its hypermethylation has been consistently reported in PanNETs with an increase in metastatic cases (204, 205, 213), and it is associated with poor prognosis in GEP-NETs (214). *MGMT* is responsible for one of the DNA damage repair mechanisms, that is, the removal of alkyl groups from guanine. Its absence causes alkylguanine to trigger a futile cycling of the mismatch repair machinery (genes *MLH1*, *MSH2*, *MSH6*, and *PMS2*) that leads to cytotoxicity (215, 216). Therefore, *MGMT* hypermethylation in PanNETs (17% to 50%) has been suggested to possibly predict response to alkylating agents such as temozolomide (204, 206, 217).

*MLH1* has been reported to be hypermethylated in 23% of 48 PanNETs, half of which were suggested to have microsatellite instability by two studies on the same cases from the same group (204, 218). However, microsatellite instability has never been confirmed in PanNET literature (20, 219, 220), although it occurs in colonic NECs (53). Only one case of PanNET has been reported in association with Lynch syndrome (221).

*TIMP3*, a tumor suppressor that inhibits matrix metalloproteinases and angiogenesis, has an uncertain role in PanNET tumorigenesis owing to contrasting reports about its methylation state. In fact, Stefanoli *et al.* (222) and Wild *et al.* (223) reported its recurrent methylation (44%), with an increase in metastatic cases (79%) and a corresponding loss of immunolabeling in tumor tissue, whereas two consecutive works by Arnold *et al.* (206, 214) reported no methylation of *TIMP3*.

Another gene involved in angiogenesis, and already known to be altered by mutation or deletion in PanNETs, is *VHL*, which has also been reported to be inactivated by methylation in 6% of cases (159).

Studies targeting methylation of several genes also investigated the prevalence of CpG island methylator phenotype (CIMP), defined as simultaneous hypermethylation of multiple CpG islands in at least two genes. CIMP was found in a large fraction of PanNETs, both functioning and nonfunctioning (52% to 100%, 83% for all PanNETs), and was associated with worse overall survival and metastasis, particularly in G2 tumors (204, 206, 222).

Hypomethylation has been shown to have a role in PanNETs as well, affecting two of the most common transposable sequences in the human genome, long interspersed element 1 (*LINE1*) and *Arthrobacter luteus* (ALU) homolog (with ALUs representing a class of short interspersed elements). Two studies have shown that PanNETs display reduced methylation of these sequences compared with adjacent nonneoplastic pancreatic tissue in 20% to 33% of cases, in correlation with higher tumor stage and poor prognosis (222, 224).

Stefanoli *et al.* (222) also detected *LINE1* hypomethylation both in cases with CIMP and in cases without CIMP, indicating that mechanisms underlying these two features are independent. The presence of hypomethylation at mobile element sites suggests the possibility of their reactivation in PanNETs, a possibility that has not yet been proven.

A recent work has exploited methylation-specific microarrays to increase the analysis throughput and investigate the existence of subgroups in a set of 60 GEP-NETs, including 35 PanNETs, 7 duodenal gastrinomas, and 18 SiNETs (18). The study showed a good although not perfect segregation of insulinomas, nonfunctioning PanNETs, and gastrinomas (both pancreatic and duodenal), whereas SiNETs clustered together with nonneoplastic samples. Despite the identification of differential methylation in many genes, the author emphasized that this was a proof-of-principle study and further validation was necessary. Moreover, methylation levels would need to be corrected considering the pattern of copy number alteration of each sample to avoid overestimation of gained regions and underestimation of loss regions.

Three of the most frequently mutated genes in PanNETs, that is, *MEN1*, *DAXX*, and *ATRX*, are known to constitute a bridge between genetics and epigenetics because of their interaction with histone methyltransferase. However, DAXX and ATRX have been also demonstrated to affect DNA methylation, with DAXX in particular binding and directing DNMT1 to the *RASSF1A* promoter (225). A recent work by Pipinikas *et al.* (226) has reported differential methylation of PanNETs harboring *DAXX* or *ATRX* mutations compared with unmutated tumors, with *DAXX*-mutated cases showing sharper differences. This observation is also interesting considering the interaction of DAXX with DNMT1 and the fact that *DAXX* mutations are twice as prevalent than ATRX mutations only in PanNETs (227, 228). However, because DAXX/ATRX impairment also leads to ALT and chromosomal instability, further investigation is required to discern the interplay between mutations, chromosomal alterations, and epigenetic changes.

#### miRNA alterations in PanNETs

Together with chromatin remodeling genes, miRNAs constitute a bridge between genetics and epigenetics. When expressed, they regulate other transcripts levels, but their expression may be altered by genetic events (mutation, copy number alterations) or by epigenetics itself (chromatin remodeling, promoter methylation). Probably also for these levels of complexity their study has lagged in recent times after a bright start, and PanNETs are no exception to this.

The first large study on PanNETs compared 12 insulinomas, 28 nonfunctioning PanNETs, and 4 acinar carcinomas to normal pancreatic tissue. All malignant tissues displayed enhanced miR-103 and miR-107, and reduced miR-155. A set of 10 miRNAs (miR-125a, -99a, -99b, -125b-1, -342, -130a, -132, -129-2, and -125b-2) could distinguish PanNETs from carcinomas, whereas insulinoma samples only differed for the overexpression of miR-204 (229). The levels of miR-21, an miRNA involved in regulation of *PTEN* and thus of the PI3K/mTOR pathway, correlated with increased proliferation (Ki-67) and the presence of liver metastasis (229).

A second study evaluated 37 PanNETs against nonneoplastic pancreas and pancreatic islets (230). Interestingly, the profiles of differentially expressed miRNAs in PanNETs were completely different when nonneoplastic pancreas or pancreatic islets were used as controls, leading to the question of which nonneoplastic cell constitutes the optimal reference for these studies. miR-193b was overexpressed in both PanNET tissues compared with pancreatic islets and sera of patients compared with healthy controls, whereas miR-642 correlated with Ki-67 and miR-210 with the presence of metastasis (230). Another study assessed the prognostic value of eight candidate miRNAs on 37 PanNETs, reporting overexpression of miR-196a as an independent predictor of earlier recurrence that also associated with higher stage, grade, and lymphatic vessel invasion at diagnosis (231).

miRNA profiles were also used to cluster murine NETs spontaneously occurring in the RipTag2 mouse model. Three groups were defined based on the presence of islet tumor signature, metastasis signature, or a combination of both [metastasis-like primary tumor (MLP)] (232). Data from the mouse model and previous miRNA (13) data were analyzed in a cross-species comparison and showed that the murine MLP signature (miR-23b, -24-1, -24-2, -27b, -132, -137, -181a1, and -181a2) is overexpressed in two thirds of human PanNETs (17). This overlap was also extended to mRNA using human data from another previous work (99). This second analysis showed that murine islet tumor and MLP clusters have a counterpart in human PanNETs, whereas a third group of human PanNETs clustered aside. The existence of a human MLP-like PanNET cluster has been confirmed also by the recent whole-genome study (20), and the transcriptional profile of this cluster showed a strong upregulation of genes under the control of the HIF1/2 factors. This opens the possibility to explore the relationship between the MLP signature and hypoxia-induced genes.

A drawback of these analyses is the lack of integration with copy number alterations that would allow connecting genetic lesions with gene expression. Moreover, for many miRNAs the list of targets is still based on software predictions that need to be improved and supported by biological validation (233).

### Pathway-based landscape of PanNET alterations

**Figure 5.**
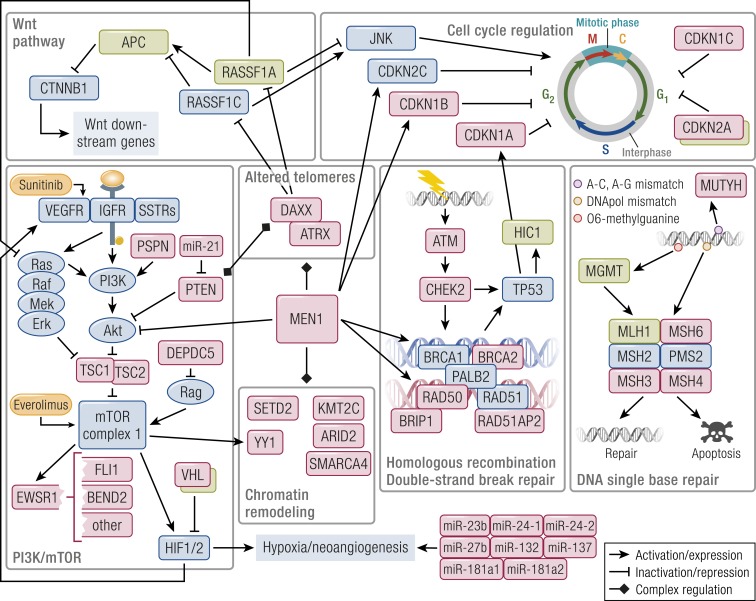
Outline of the main altered pathways in PanNETs. Pathway members whose genetic alteration has been proven are shaded in red, and those inactivated by epigenetics are in green. Approved targeted drugs are shaded in orange. *MEN1* interacts and modulates all core pathways acting as a hub gene. DAXX/ATRX also cooperate with the other genes of the chromatin remodeling complexes. DAXX fosters RASSF1A promoter methylation, but also retains RASSF1C in the nucleus, releasing it upon DNA damage; it also modulates PTEN distribution between the nucleus and the cytoplasm whereas PTEN modulates DAXX’s gene expression regulation. MGMT repairs of *O*^6^-methylguanine but, in case the repair fails, it triggers futile cycling of the mismatch repair complex that leads to cytotoxicity. Both the mTOR pathway and a set of dysregulated miRNAs trigger HIF1/2-dependent gene expression in a large subset of PanNETs. The role of chromatin remodeling genes is complex, still unclear, and under active investigation.

The recent whole-genome sequencing study has pinpointed the main pathways involved by alterations at the genomic level, reconciling data from the previous, smaller scale studies, and adding relevant insights (20). Four are the altered pathways emerging from this work: DNA damage repair, chromatin remodeling, telomere alteration, and the PI3K/mTOR signaling pathway. Genes altered in these pathways are further clarified by the literature on epigenetic alterations. The latter also add information about the deregulation of two additional molecular routes, namely the Wnt and cyclin-dependent proliferative pathways (Fig. 5).

DNA damage repair mechanisms were altered by both germinal (*MUTYH*, *CHEK2*, *BRCA*) and somatic mutations. These mutations involve both single-base repair systems, where *MGMT* hypermethylation also plays a role, and the HR double-strand break repair. Both have been associated with therapeutic options, namely the use of alkylating agents such as temozolomide in MGMT-deficient PanNETs and platinum or poly(ADP-ribose) polymerase (PARP) inhibitors in the case of HR-deficient tumors (217, 234).

The chromatin remodeling compartment is the less understood at the moment, given the broader and more indirect effect exerted by its altered members. The impact of *ATRX*/*DAXX* alteration was also shown to go beyond the ALT phenomenon, involving methylation or direct regulation of genes such as *RASSF1* and *PTEN* (182, 225).

The mTOR pathway involvement is sustained by transcriptional data, with *TSC2* and *PTEN* downregulation as prominent events in a large fraction of PanNETs. This downregulation was associated with poor outcome (99) and mutation of several genes, most of which (*TSC1/2*, *PTEN*, *DEPDC5*, *VHL*) inhibit the mTORC1 complex or its downstream activation of *HIF1/2*. Activation of HIF1/2 downstream genes has been linked to a miRNA signature expressed by tumors prone to metastasis, but the role of these miRNAs still needs elucidation (17, 20). The pathway may be further enhanced by methylation-mediated suppression of *RASSF1A* (202).

Suppression of *RASSF1A* and *APC* also sustains the Wnt pathway, although the cyclin-dependent cell cycle program may be dysregulated by loss of the RASSF1A/RASSF1C balance. Mutations involving the CDKN family of inhibitors, or methylation of *CDKNA2*, may contribute to this pathway deregulation as well (20, 204, 205, 213).

Even when they are not directly altered, *MEN1* loss may deregulate components of the HR DNA repair machinery, of the mTOR pathway (Akt), or members of the cyclin-dependent kinase inhibitors such as *CDKN1B* and *CDKN2C* (141–144). Given its broad range of interaction with histone methylase complexes, *MEN1* acts similar to a “hub” gene whose loss may alter many involved pathways in a pro-oncogenic way.

Despite that most alterations converge in a relatively small number of pathways, some remarks are important. Most events happen at a relatively low frequency, with the exclusion of *MEN1* and *DAXX*/*ATRX* mutation, and of *RASSF1A*, *HIC1*, and *APC* methylation. Therefore, data integration is mandatory in future studies to understand which grade of each pathway alteration occurs in different cases. A partial integration has been proposed in the recent whole-genome study and demonstrated that cases with *DAXX*/*ATRX* alteration and ALT phenotype often display recurrent chromosomal loss. Conversely, cases with germline mutations in DNA repair genes tend to lack *MEN1* biallelic loss. Given these results, extending this approach to methylation and transcriptomics data seems to be the right direction for the future.

The second remark concerns lack of high-throughput methylation data on PanNETs, which could be integrated with mutations and chromosomal alteration similarly to what Karpathakis *et al.* (19) recently did with SiNETs. Finally, the functional exploration of chromatin remodeling genes will require ample investment and disease models. At present, the only available one seems to be the RipTag2 mouse, as the attempts to establish patient-derived xenografts have been unsuccessful to date (17). This is a hurdle that has to be overcome.

### Genetic and epigenetic alterations of SiNETs

#### Genetic alterations of SiNETs

Differently from pancreatic NETs, for a long time SiNETs have been considered to be exquisitely sporadic. The only notable exclusions are duodenal somatostatinomas and gastrinomas, which constitute a minor portion of SiNETs and were recently reported to be molecularly more similar to their pancreatic counterpart (18, 213).

The recently identified familial SiNETs still lack a driver gene, although it seems to overlap with its sporadic counterpart regarding clinical behavior and chromosomal alterations. In particular, the latter often include loss of chromosome 18 in primary tumors and gain of chromosome 7 in metastases (73).

The largest fraction of SiNETs arises in the ileum/cecum, and several mutational or cytogenetic studies have been recently performed on this tumor type. However, mutational studies to date are conflicting, and the lack of a driver alteration such as *MEN1* for PanNETs is the most striking feature. This is probably not due to differences in mutation rates, as these are similarly low in SiNETs (0.77 mutations per megabase) and in PanNETs (0.82 mutations per megabase). Nonetheless, rates of nonsilent exonic mutations of SiNETs are lower (0.1 per megabase vs 0.3 per megabase) than in PanNETs (15, 16, 40).

The first whole-exome sequencing study identified as few as 211 nonsilent mutations with no recurrently mutated genes (15). Somatic copy number alterations were detected, showing recurrent loss of chromosome 11 and 18 and gain of chromosome 4, 5, 14, and 20, as previously reported by CGH studies (72, 73, 170). An integrated analysis showed that alterations were dominated by CNVs and converged toward deregulation of two pathways: TGF-*β*/Wnt (*SMAD4* copy loss) and especially PI3K/mTOR (*AKT1* plus *PDGFR* or *AKT2* plus *MTOR* plus *PIK3CD* copy gain).

A second study by Francis *et al.* (16) performed a mix of whole-genome and whole-exome sequencing on 50 cases, including 39 primary tumors and 19 metastases. This study confirmed the previous landscape of CNVs but also reported recurring mutations (8%) and locus deletions (14%) of the *CDKN1B* gene involved in negative regulation of the cell cycle (16). The gene was therein suggested to be haploinsufficient, and the mutation rate was soon confirmed by further studies (19, 235). As for chromosomal alterations, loss of chromosome 18 was the main event (76%), although gains of chromosomes 4, 5, 14, and 20 appeared simultaneously in 26% of cases. Considering that germline *CDKN1B* mutations characterize patients with MEN4, that mutations in *MEN1* affect *CDKN1B* expression, and that animal models supported *CDKN1B* haploinsufficiency, its alteration (including locus deletions) may be a driver event in up to 20% SiNETs (149–155). However, intratumor heterogeneity for *CDKN1B* mutations has been reported as well, which suggests further evaluation (71).

*APC* has also been reported to be frequently altered in SiNETs, but this finding still needs independent validation. Bottarelli *et al.* (71) found high prevalence (23%) of *APC* mutations by targeted sequencing of 30 ileal SiNETs. Moreover, LOH at the *APC* locus affected 15% of cases, including two of the seven mutated tumors (71). This is a well-known tumor suppressor gene, which negatively regulates the Wnt pathway by targeting *β*-catenin for degradation. As already remarked, its loss of function is linked not only to proproliferative effects but also to chromosomal segregation defects (211). Similar to *CDKN1B*, *APC* is a haploinsufficient gene and has also shown to be affected also by milder alterations of its function (211). Interestingly, mutations identified by Bottarelli *et al.* were novel, suggesting that NETs may arise from a different subset of *APC* alterations than those found in carcinomas. However, because only one *APC* mutation was reported by Francis *et al.* (16) and 4 of 52 cases by Simbolo *et al.* (236), further studies are necessary to validate the real prevalence of these alterations as has happened for *CDKN1B*.

The existence of a mitotic segregation defect along SiNET tumorigenesis was supported also by the fact that focal CNVs and rearrangements in these tumors are rare, whereas the gain or loss of whole chromosomes is frequent and tend to cluster. Moreover, CNVs tend to increase in metastases compared with primary tumors (72). The co-occurrence of CNVs was shown also by Francis *et al.* (16) and further confirmed by Karpathakis *et al.* (19), who performed a clustering analysis and described the existence of three groups (Fig. 4). Group 1 was affected by chromosome 18 copy loss and contained all *CDKN1B* mutated cases, group 2 lacked any specific alterations, and group 3 showed multiple copy gains at chromosomes 4, 5, 14, and 20. The copy number profile also affected methylation profiles especially in group 3, and disease-free survival was progressively poorer moving from group 1 to group 3 (16, 19).

The report of chromosome 18 losses led to the investigation of two of the most appealing tumor suppressor genes residing therein, *SMAD2* and *SMAD4*. However, these were shown to be unaffected by several studies; other candidates were also investigated with no success (75, 237, 238). Only recently, a study associated loss of *SMAD4* at immunohistochemistry (11 of 38 cases) with poor prognosis after resection in a mixed cohort of gastrointestinal NETs, 60% of which were SiNETs. However, comparing the high frequency (75%) of chromosome 18 loss and the lower (29%) frequency of cases with loss of protein expression, it is probable that an independent second hit has to occur (239).

#### Epigenetic alterations of SiNETs

Although their genomic landscape is radically different, both PanNETs and SiNETs showed hypermethylation of the *RASSF1A* promoter, with SiNETs affected to a lower degree (76, 78). A first study on a panel of genes reported promoter hypermethylation of only two genes in SiNETs, namely *RASSF1A* and *CTNNB1.* This methylation pattern was absent from both normal enterochromaffin cells and appendiceal NETs. Moreover, methylation levels were higher in metastases compared with primary tumors and inversely correlated with gene expression (76).

These data were substantially confirmed by a larger study, which also reported methylation and expression changes in regional and distant metastases (78). Expression of the two genes increased in regional metastases while decreasing to the level of primary tumors or lower in distant metastases. Conversely, methylation levels compared with those of primary tumors were variable in regional metastases and threefold higher in distant metastases. Low levels of *RASSF1A* expression were also associated with poorer prognosis (78). The study also analyzed *LINE1* methylation and found a correlation between *LINE1* hypomethylation and loss of chromosome 18. Those findings confirmed a previous study by Choi *et al.* (224), who demonstrated that *ALU* and *LINE1* hypomethylation in SiNETs was more extensive than in PanNETs and correlated with chromosome 18 copy loss and hypermethylation of *RASSF1A* and *MGMT* promoters. *LINE1* hypomethylation was confirmed by a third study, which also reported no correlation with tumor grade or stage (240).

Chromosome 18 was again involved by the work of Edfelt *et al.* (241), who investigated *TCEB3C.* This gene encodes for elongin A3, the only imprinted gene residing on chromosome 18. Expression of elongin A3, which is positive in normal mucosa, was very low/undetectable in 77% of tumors; as expected, 89% had only one copy of the gene. A subset of cases were analyzed for CpG methylation, and *TCEB3C* promoter was found to be methylated in all, whereas *in vitro* experiments suggested a double level of regulation by both DNA and histone methylation. Moreover, *de novo* expression of *TCEB3C* reduced cell proliferation *in vitro*. Elongin A3 is involved in RNA transcription, but its role as a tumor suppressor needs further investigation.

The *SEMA3F* gene, encoding semaphorin 3, was also recently studied by immunohistochemistry and methylation analysis in 101 SiNETs. Loss of protein expression was reported in 50% of tumors and correlated with higher tumor stage, proliferation rate, and promoter methylation. Functional *in vitro* experiments suggested an involvement of semaphorin 3 in the inhibition of the PI3K/mTOR pathway (242).

*UCHL1*, a tumor suppressor gene that stabilizes p53 (243), was investigated in a group of 46 GEP-NETs as a candidate derived from preliminary transcriptome data. Its expression was virtually absent and correlated with promoter hypermethylation in metastatic cases, 70% of which were SiNETs. Despite these promising results, the low number of non-SiNET cases prompts further studies to determine whether *UCHL1* deregulation is an event involving most GEP-NETs or specific subtypes (244).

Methylation has also been investigated by high-throughput studies, one of which demonstrated differential methylation among 10 SiNETs and their matched metastases, with global levels decreasing in the latter (79). Despite this, the aggressiveness of tumors in this study correlated with higher methylation in the primary site. Among the most hypermethylated genes, the presence of *TCEB3C* confirmed previous findings, although the other targets require further verification (79).

*“The consistent activation of mTOR pathway has been demonstrated by a recent integrated genomic/ epigenomic analysis…”*

The largest genome-wide study to date involving methylation was performed by Karpathakis *et al.* (19) on 97 SiNETs, and has been cited above as the first study integrating CNV profiles, mutation of *CDKN1B*, and methylation array data (SiNETs panel of Fig. 4). As for methylation, the most striking report in the study concerns the high number of hypermethylated genes in the group of tumors with multiple copy gains. Alternatively, the other two groups had similar profiles, and their pattern of differentially methylated genes appeared to be complementary to that of the first. Comparison with normal tissue produced a long list of differentially methylated genes, which await further validation. In the meantime, enrichment analysis reported prominent involvement of the MAPK, mTOR, and Wnt pathways. The same pathways also emerged as differentially deregulated from a second enrichment analysis across the three identified SiNET groups (19).

In a follow-up study, the same authors compared methylation profiles of primary SiNETs and metastases. Using the same list of candidate genes produced in the previous work, they showed progressive differential methylation in 67% of genes. Progressive differential expression was detected in only 24% of these genes, although the percentage rose to 51% considering a false discovery rate of 0.1, which is common in high-throughput studies (21). Although the authors claimed that progression to metastasis could be epigenetically driven, they also confirmed previous data regarding progressive accumulation of CNV in metastases compared with primary tumors. This leads to the question of how CNV and methylation profiles influence each other and which interactions are critical to this mutual influence.

Another open question concerns the role of chromatin remodeling in SiNETs. Only sparse mutations have been detected in chromatin remodeling genes to date, as well as in only small studies focusing on chromatin-involved SiNETs. Warneboldt *et al.* (245) investigated the expression of histone H1x, a histone that stabilizes heterochromatin, and detected increased levels of H1x in tumor compared with normal tissue. Conversely, Magerl *et al.* (246) detected almost ubiquitous (93%) high levels of histone 3 dimethylation at lysine 4, an important modification that was associated with active transcription and better prognosis in other tumor types. These data, together with the above report on regulation of the *TCEB3C* gene (241), show that some chromatin regulations do happen in SiNETs, and this needs to be explored.

#### miRNA alterations of SiNETs

Studies involving miRNA profiling of SiNETs mainly focused on the differences between localized and metastatic disease, with the latter constituting >70% of all diagnosed cases (21). Four studies reported an interesting overlap of differentially expressed miRNAs. The first of these reports was produced by Reubel *et al.* (247), who profiled 95 miRNAs in eight cases with matched primary tumors and metastases. Upregulation of miR-19a, -183, and -488 and downregulation of miR-10b, -133a, -145, -146, and -222 were detected in metastases; validation was also performed for miR-133a in an independent cohort. This latter miRNA is transcribed with miR-1 from two loci, one on chromosome 18q and the other on chromosome 20q. They are part of the so-called “canonical myomiRs” (miR-1, -133a, -133b, and -206) involved in key regulation of muscle, brain, nerve, and adipose structures. Deregulation of these miRNAs has been reported in a large number of cancers, including pancreatic ductal adenocarcinoma but not PanNETs (248).

A second report by Li *et al.* (249) compared miRNA profiles of five primary SiNETs with matched mesentery and liver metastases and validated the results in a second cohort of three cases. Upregulated miRNAs were miR-96, -182, -183, and -196a, whereas downregulation affected miR-31, -129-5p, -133a, and -215, thus confirming the previously reported upregulation of miR-183 and the downregulation of miR-133a.

A third report by Miller *et al.* (250) analyzed 28 SiNETs and matched normal tissue (n = 14), lymph node metastases (n = 24), and liver metastases (n = 15). The analysis identified 39 differentially expressed miRNAs, with good overlap of upregulated miRNAs in primary tumors or metastases compared with normal tissue. Results substantially confirmed the previously described pattern of upregulated and downregulated miRNAs, with the addition of upregulated miR-204, -7-5p, and -375 and downregulated miR-1 and miR-143-3p. In particular, downregulation of miR-1 was expected from previous experiments, because it is transcribed with miR-133a as a single cistron.

The overlap of deregulated miRNA between SiNETs and PanNETs was limited to only two overexpressed miRNAs: miR-204 was specific for insulinomas (13), whereas miR-196a was an independent predictor of poor prognosis (231). The latter miRNA has been further studied *in vitro*, and results demonstrated that it is a negative regulator of many genes. These include *HOXA9* and *HOXB7*, two homeobox genes upregulated in other cancers, and genes of the Wnt pathway, including *CTNNB1*, *FZD5*, *LRP4*, *LRP5*, *LRP6*, and *RSPO2* (251).

These data, together with the previously reported hypermethylation of *CTNNB1* gene, suggest that the Wnt pathway may be suppressed in SiNETs. However, given the broad range of targets and concurrent alterations, more investigation is warranted. Of note, miR-196a locus maps on chromosome 17q, which is amplified in metastatic SiNETs and harbors the proto-oncogenic *HER2/neu* locus.

Several consistently reported miRNAs were also investigated as serum biomarkers for comparison of patients with SiNETs with healthy subjects. Downregulation of miR-31, -129-5p, -133a, and -215 was consistently detected in sera of patients with or without metastatic disease. Alternatively, strong upregulation of miR-196a and miR-200a was observed only in patients with liver metastasis, whereas miR-96 and miR-182 upregulation was weak or absent. Treatment with somatostatin analogs caused a strong upregulation of miR-96, -182, -196a, and -200a in patients of all stages, although downregulated miRNAs were substantially unaffected (252). Although the biological meaning of these data is still unclear, these miRNA-based assays may prove useful in the follow up of patients, in the absence of a gene mutation panel such as those recently proposed for lung cancer patients (253).

#### Pathway-based landscape of SiNET alterations

**Figure 6.**
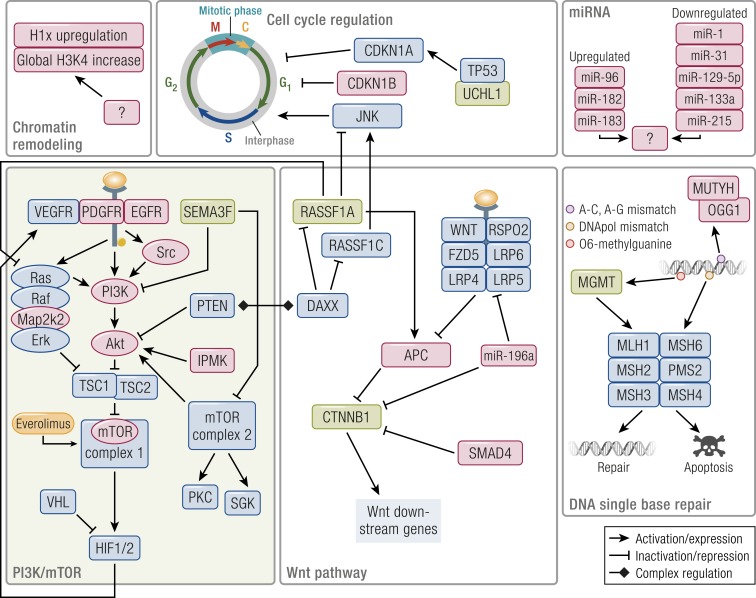
Outline of the main altered pathways in SiNETs. Pathway members whose genetic alteration has been proven are shaded in red, and those inactivated by epigenetics are in green. Approved targeted drugs are shaded in orange. The mTOR pathway is detected to be altered mainly via gene amplifications (*PIK3CD*, *AKT1*, *mTOR*, *SRC*, *MAP2K2*, *PDGFR2*) and epigenetically by hypermethylation of a large set of genes [represented by the green shade, described in Karpathakis *et al.* (19, 21)]. *RASSF1A* downregulation by hypermethylation and *SMAD4* loss may foster the Wnt pathway, which is however contrasted by CTNNB1 methylation and miR-196a hyperexpression, with the latter repressing transcription of Wnt pathway genes. The cell cycle is involved in CDKN1B-mutated tumors whereas UCHL1, a TP53 stabilizer, is lost in metastatic tumors by methylation. *MUTYH*, *OGG1*, and *IPMK* mutations involve familial cases, whereas the role of miRNA and chromatin deregulation is currently unclear.

Despite the efforts recently expended on the genomic exploration of this tumor type, the overall pathway landscape for SiNETs is less clear than that of PanNETs. Nonetheless, it again involves mTOR pathway, cyclin-dependent cell cycle regulation, the Wnt pathway, and DNA single-base repair (Fig. 6). The relatively low prevalence of mutations is compensated by recurrent gene copy number gains affecting known oncogenes. Moreover, mutations or copy losses of genes that are known to be haploinsufficient, such as *APC* and *CDKN1B*, may not need a second hit to exert their effect (150, 211).

The consistent activation of mTOR pathway has been demonstrated by a recent integrated genomic/epigenomic analysis by Karpathakis *et al.* (19, 21), who suggested that it may be driven by the amplification of *EGFR*, *HER2/Neu*, or *PDGFR*. In fact, the enrichment profile of hypermethylated genes pointed to activation of these receptor kinases, whose genes were amplified in metastatic cases. Their signaling pathways converge to feed the mTORC1 complex through Ras, PI3K, or Src (19, 21). Inactivation of semaphorin 3 by methylation also moves in this direction, releasing the inhibition of both PI3K and mTORC2. The latter is less sensitive than mTORC1 to the approved targeted drug everolimus and may drive resistance to therapy (242).

Cell cycle regulation may be the second driving force of SiNETs; this has been suggested mainly due to the alteration of *CDNK1B* by mutation in 8% to 10% of cases and copy loss in 14% (16). Hypermethylation of *RASSF1A* may contribute as well by releasing the inhibition of JNK, whereas loss of *UCHL1* expression may destabilize p53 and thus its downstream effector *CDKN1A* (243, 244). This deregulation of cell cycle checkpoint seems to be alternative to mTOR hyperactivation. In fact, as reported by Karpathakis *et al.* (19, 21), cases with *CDKN1B* mutations usually display chromosome 18 loss and lack chromosome 4, 5, 14, and 20 copy gains. As a consequence, they also lack the associated mTOR pathway enhancement and display a better outcome.

The Wnt pathway in SiNETs is affected by a mixture of alterations that may result either in promotion or in suppression of its signaling. Perhaps for this reason, its role in the overall landscape is probably less clear. Indeed, mutations in *APC* and repression of *RASSF1A* by hypermethylation should release *β*-catenin by repression and foster its transcriptional activity. In the same direction, the loss of *SMAD4* may remove another level of negative control on *β*-catenin (239, 254). Despite this, the promoter of the *CTNNB1* gene resulted frequently hypermethylated and expression of *β*-catenin was concomitantly reduced in metastases (76, 78). This further correlates with enhanced levels of miR-196a in SiNET liver metastases, as this miRNA was associated with reduced expression of several Wnt pathway members, including *CTNNB1*, *FZD5*, *LRP4*, *LRP5*, *LRP6*, and *RSPO2* (249, 251). Further experiments and integrated analyses are necessary to understand whether subgroups of tumors display concurrent alterations, whether this concurrence of alterations leads to any effective downstream activity, or whether downregulation of the Wnt pathway is just a feedback reaction to its signaling activation. This latter hypothesis was partly supported by the fluctuation of RASSF1A in matched primary tumors, lymph node metastases, and liver metastases (78), which makes for an interesting starting point.

DNA repair alterations are relatively rare in SiNETs, although germline mutation of *MUTYH* and *OGG1* have been recently found in both familial and sporadic cases. However, the estimated frequency of these mutations in sporadic SiNETs is 1% to 2%, and there are no other significant reports of alterations in the single-base repair or HR double-strand break repair (219). Alternatively, hypermethylation of *MGMT* has been detected (224). Interestingly, this feature correlated with *RASSF1A* hypermethylation, global hypomethylation at *ALU* and *LINE* sites, and loss of chromosome 18 (224), although the biological meaning of this context still needs to be elucidated.

At present, the mTOR pathway emerges as the central node of the aggressiveness of SiNETs: its activity is supported by recent and integrated studies that confirmed its further activation in metastases compared with primary tumors. The other pathways seem to be either affected in a minor fraction of cases with better outcome (*CDKN1B*, cell cycle) or not yet well defined (Wnt, DNA repair). miRNA and chromatin deregulation have been associated with metastatic behavior as well, but the connection with other molecular alterations and defined SiNET subtypes requires active investigation.

### A pathway-based comparison of PanNETs and SiNETs

The molecular differences between PanNETs and SiNETs have been progressively supported by scientific reports, with an important premise: most molecular data on SiNETs focused on the more prevalent ileal SiNETs. In contrast, NETs of the duodenum/jejunum are biologically closer to PanNETs. In particular, gastrin-expressing duodenal NETs are often associated with MEN1 and show a global methylation profile similar to pancreatic gastrinomas (18, 59, 61). Moreover, duodenal NETs often harbor *CTNNB1* mutations, which are virtually missing from ileal SiNETs (255). This observation also emphasizes how anatomic origin may influence the molecular alterations of these tumors.

The molecular divergence of PanNETs and SiNETs was already suggested by early cytogenetic and CGH studies, and later genome-wide analyses confirmed this idea (Fig. 4). Indeed, alteration patterns are almost completely nonoverlapping. However, both tumor types include a group with specific loss of one chromosome (11 for PanNETs, 18 for SiNETs) and a group with multiple copy gains. Alternatively, SiNETs do not feature the polyploid and RPCL groups, but they comprise another group lacking any characteristic chromosomal alterations (19, 20).

These differences are also consistent in a pathway-based comparison (Fig. 5*vs*Fig. 6). The hallmark of both NET types is the activation of the mTOR pathway, but this is achieved through different genetic routes. PanNETs feature mutational inactivation of mTORC1-repressing genes, whereas SiNETs display copy gain of mTOR itself and the activation of receptor or cytosolic kinases. SiNETs also feature methylation-induced silencing of genes involved in mTOR, MAPK, and Src signaling, information that is not yet available for PanNETs. Similarly, PanNETs with an aggressive transcriptional profile display the enrichment of genes controlled by the hypoxia factors HIF1/2, but this information is not available for SiNETs (19–21).

*MEN1* and *DAXX*/*ATRX* alterations are specific to PanNETs, although information on chromatin remodeling and telomere biology is scarce for SiNETs.

As for the Wnt pathway, partial activation may exist in both tumor types. However, despite that miR-196a upregulation has been described in both SiNETs and PanNETs, with prognostic implications in the latter, the modulation of *CTNNB1* and its downstream partners has been shown in SiNETs but not in PanNETs.

DNA repair seems to be involved more in PanNETs than in SiNETs, with the former displaying alterations affecting multiple members of single-base repair complexes and the exclusive involvement of double-strand break repair (20). Conversely, reported SiNET alterations involved only single-base repair and to a lesser extent (133, 224).

Both tumor types involve the alteration of the cell cycle, albeit the implicated genes are different: SiNETs more frequently involve *CDKN1B* mutation or copy loss, whereas this locus is rarely affected in PanNETs with the exception of patients with MEN4. *RASSF1A* inactivation may alter the cell cycle in both tumor types, whereas hypermethylation of *CDKN2A* and inhibition of *CDKN1B* and *CDKN2C* caused by *MEN1* loss are characteristic of PanNETs (141, 204, 205).

Although many details need further exploration and the level of chromatin remodeling has just begun to be uncovered, comparison of the two overall landscapes suggests that mTOR pathway activation is a main event in both SiNETs and PanNETs. The latter aldo display concurrent involvement of cell cycle and Wnt pathways. The comprehensive results reported by most recent studies on both categories of NETs show that integration of large-scale molecular analyses is a necessary strategy to determine the degree of alteration of each pathway in each single tumor.

## Advances in Targeted Therapy and Detection of GEP-NETs in Light of Recent Molecular Findings

A great deal of effort has been applied to the development of targeted therapies in the recent years; NETs have benefitted from this, as the mTOR inhibitor everolimus and the tyrosine kinase inhibitor sunitinib were recently approved for targeted therapy of GEP-NETs (256, 257).

However, the above treatment options face low response rate and resistance phenomena in both the short term and long term, which limit their efficacy in prolonging the time to progression (258–264). Consequently, the latest preclinical and clinical reports have investigated the combination of molecular drugs to address this problem (261, 262, 265–267). In particular, studies on cell lines showed promising results by combination of everolimus and PI3K inhibitors. The latter overcame long-term resistance mediated by glycogen synthase kinase-3 and restored everolimus sensitivity (184, 261, 262). These results, however, still need to be validated in a clinical setting.

Recent reviews have pinpointed that clinical trials on GEP-NETs need to be improved by exploiting molecular markers derived from the accumulated knowledge on altered pathways. These may help patient stratification and possibly predict response to therapy (23, 33). Moreover, according to the data summarized in the present review, the knowledge of differences in pathway alterations between NET types and between single cases is going to become fundamental to devise the best combination of treatments.

Given their low incidence and heterogeneity, GEP-NETs often have been studied together, and the initial notion about their molecular imbalances were somewhat generic and confusing. Data obtained studying more homogeneous cohorts by high-throughput assays confirmed a discrepancy between the high prevalence of transcriptional or epigenetic deregulation in some pathways and the relatively low frequency of genetic events affecting selected members of the same pathways. For instance, the mTOR pathway was affected in 80% of PanNETs as assessed from *TSC2* and *PTEN* expression (99), but *TSC2* and *PTEN* mutations were reported in 9% and 7% of PanNETs, respectively (14, 20).

The progressive integration of genetics and epigenetics data, especially those converging in pathways that may be addressed by targeted therapies, should clarify which molecules may be used as reliable biomarkers. Other important information that this data integration may yield is the anticipation of resistance mechanisms that are potentially targetable by combination therapies.

An example for SiNETs may be drawn from Fig. 6: data derived from current literature foresee a concurrent activation of both mTORC1 and mTORC2 complexes, with the latter being insensitive to everolimus. This activation is fostered by both loss of semaphorin 3 and amplification of genes encoding for members of the Src and Ras-MAPK pathways. This is a general scenario, but knowledge of the actual hyperactivated routes in each patient might allow anticipation of resistance mechanisms before their onset and planning a better therapeutic strategy.

Many are the drugs under preclinical assessment or in phase 1 trials, including PI3K inhibitors, dual mTOR inhibitors, or multikinase inhibitors targeting both tyrosine kinase receptors and cyclin-dependent kinases (268). The experimental approaches to validate their use on GEP-NETs should take into account the existence of a large diversity in alterations of the very same pathways, possibly leading to different response to similar treatments.

*“…measurement of specific RNAs in the blood of patients with GEP-NETs are being actively developed.”*

Meanwhile, the combination of mTOR inhibitors with VEGFR inhibitors, endothelial growth factor receptor (EGFR) inhibitors, or MAPK kinase inhibitors gave encouraging preclinical results and deserve further exploration, but this will require a careful subclassification of candidate patients based on their actual pathway deregulation to have a chance of success. In this scenario, most of the used cell lines proved to be closer to NECs than to NETs from the molecular point of view (29–31). Therefore, molecular subtypes of tumors produced by mouse models that were recently correlated to similar subtypes in human PanNETs (17, 20, 32) may be the best available tool to assess the variability in the response to a different therapeutic approach.

A better integration of genetic, epigenetic, and transcriptomic data may also benefit the development of companion diagnostics and the set-up of multianalyte tests. These assays have been recently proposed to detect, characterize, and predict disease status of GEP-NETs by measuring specific mRNAs or miRNAs in the blood of patients with GEP-NETs (233, 269, 270). These tests seek to overcome the limitations of current methods for disease assessment, limited to CT/MRI or functional/somatostatin-based imaging techniques and blood measurement of chromogranin A, serotonin, and pancreastatin (271, 272). Moreover, these tests might also prove useful in predicting responses to standard treatments such as somatostatin analogs, radionuclide therapy, or ablation procedures. These potentialities have suggested the use of multianalyte tests in ongoing clinical trials (33). Moreover, alternative versions of these multianalyte blood approaches could take advantage of the recent and upcoming integrated analyses that are defining GEP-NET molecular subtypes. The above-reported blood tests for GEP-NETs have been relying on RNA, whereas the information on circulating tumor DNA (ctDNA) is still scarce. The lack of recurrent mutations makes it difficult to use ctDNA as a diagnostic tool for GEP-NETs. A recent case report suggested that it would be more feasible to sequence a tissue sample from the tumor and then design a personalized ctDNA blood-based or even urine-based assay (273). The latter could be applied to monitor the disease, as recently demonstrated by Boons *et al.* (274), who used this approach to correctly identify three metastatic cases in a set of 10 patients with PanNETs. Another promising approach has been proposed for PanNETs, exploiting whole-exome sequencing on ctDNA (275). Data were very preliminary and the concordance of alterations between tissue DNA and ctDNA was variable. Nonetheless, ctDNA sequencing with large gene panels has the potential to uncover genetic alterations linked to targeted therapies, as demonstrated on other NENs (276, 277). More research is necessary to understand whether ctDNA will be useful for GEP-NETs and to what extent.

## Summary of Our Knowledge on GEP-NETs, Current Blind Spots, and Future Developments

If one word can define GEP-NETs it is “heterogeneity.” Although they have been grouped under a common denomination, they display more differences than similarities. Tumors from different locations exhibit a different relationship with genetic or hormone-derived syndromes, display different driver alterations in sporadic cases, have a different ratio of aggressive *vs* indolent cases, and often have extremely different outcomes.

Despite this, they share some similarities when compared with carcinomas, leading to a less dramatic deregulation of the cell cycle. This is not driven by loss of *RB1* and *TP53* but rather by modulation of the *CDKN* gene family. Similarly, proliferation is usually not sustained by mutation of *KRAS*, but rather by loss of mTOR pathway inhibitors (*MEN1*, *PTEN*, and *TSC2* for PanNETs) or by amplification of genes encoding members of the Ras/MAPK, Src, or PI3K/Akt pathways, which foster mTOR signaling in SiNETs.

These alterations have a low frequency and thus do not occur in each tumor. In fact, different groups of alterations cluster in different groups of tumors, providing varying levels of aggressive potential. Although these observations have been produced by analysis of PanNETs and SiNETs, they can fuel research on the remaining neglected GEP-NET types. However, it is expected that GEP-NETs of other locations will also display peculiar alterations or variations on the theme of mTOR and cell cycle signaling deregulation.

Although much information has been produced in PanNETs and SiNETs, the integration of these data has just begun and the key molecular nodes are still far from being completely understood. Especially for the chromatin remodeling compartment, experiments on disease models will be vital to understand how changes in histone regulation are affected by genetic events, and which events lead to the peculiar chromosomal alterations that have been reported. Disease models, especially those producing a similar heterogeneity to that observed in humans, will be similarly vital to understand if and how the differences in modulation of the same pathway can predict resistance mechanisms to targeted drugs.

Another theme that has been marginally investigated is intratumor heterogeneity of GEP-NETs. Studies published in most recently have reported the existence of low-abundance alterations, including both mutations and copy number changes (184, 278). This deserves further investigation, especially considering their potential role in tumor progression or resistance to therapy.

Equally underdeveloped and important for patient management is the theme of longitudinal sampling, although the first efforts can be seen. A work published this year presented the U-CAN project of the Uppsala and Umea Universities to achieve longitudinal collection of blood, tissues, and multidisciplinary clinical data from patients with cancer, including NETs (279). Similarly, multianalyte blood-based tests using the measurement of specific RNAs in the blood of patients with GEP-NETs are being actively developed (233, 269, 270), although the detection of circulating tumor DNA in the blood or urine is still in an early phase (273, 275–277).

In this framework, the widespread use of high-throughput technologies for producing information and the development of tailored computational methods to simplify their analysis is expected to be of paramount importance.

## Abbreviations

ALT: alternative lengthening of telomeres
ALU: *Arthrobacter luteus*
CGH: comparative genomic hybridization
CIMP: CpG island methylator phenotype
CNV: copy number variation
ctDNA: circulating tumor DNA
G1: grade 1
G2: grade 2
G3: grade 3
GCC: goblet cell carcinoid
GEP: gastroenteropancreatic
HIC: hypermethylated in cancer
HIF: hypoxia-inducible factor
HR: homologous recombination
JNK: c-Jun N-terminal kinase
LINE1: long interspersed element 1
LOH: loss of heterozygosity
MAPK: mitogen-activated protein kinases
MEN: multiple endocrine neoplasia
MEN1: MEN type I
MEN4: MEN type IV
MLP: metastasis-like primary tumor
mTOR: mammalian target of rapamycin
NEC: neuroendocrine carcinoma
NEN: neuroendocrine neoplasm
NET: neuroendocrine tumor
NF1: neurofibromatosis type I
PanNEN: pancreatic NEN
PanNET: pancreatic NET
PDGFR: platelet-derived growth factor receptor
PI3K: phosphatidylinositol 3-kinase
Ras: rat sarcoma
RPCL: recurrent pattern of chromosomal loss
SiNEN: small intestine NEN
TS: tuberous sclerosis
VEGFR: vascular endothelial growth factor receptor
VHL: von Hippel–Lindau
WHO: World Health Organization
